# Metal Recovery from Natural Saline Brines with an Electrochemical Ion Pumping Method Using Hexacyanoferrate Materials as Electrodes

**DOI:** 10.3390/nano13182557

**Published:** 2023-09-14

**Authors:** Sebastian Salazar-Avalos, Alvaro Soliz, Luis Cáceres, Sergio Conejeros, Iván Brito, Edelmira Galvez, Felipe M. Galleguillos Madrid

**Affiliations:** 1 Centro de Desarrollo Energético de Antofagasta, Universidad de Antofagasta, Av. Universidad de Antofagasta 02800, Antofagasta 1240000, Chile; sebastian.salazar@uantof.cl; 2 Departamento de Ingeniería en Metalurgia, Universidad de Atacama, Av. Copayapu 485, Copiapó 1530000, Chile; alvaro.soliz@uda.cl; 3 Departamento de Ingeniería Química y Procesos de Minerales, Universidad de Antofagasta, Av. Universidad de Antofagasta 02800, Antofagasta 1271155, Chile; luis.caceres@uantof.cl; 4 Departamento de Química, Universidad Católica del Norte, Av. Angamos 610, Antofagasta 1270709, Chile; sconejeros@ucn.cl; 5 Departamento de Química, Facultad de Ciencias Básicas, Universidad de Antofagasta, Av. Universidad de Antofagasta 02800, Antofagasta 1240000, Chile; ivan.brito@uantof.cl; 6 Departamento de Ingeniería Metalúrgica y Minas, Universidad Católica del Norte, Av. Angamos 610, Antofagasta 1270709, Chile; egalvez@ucn.cl

**Keywords:** metal recovery, mixing entropy battery, Prussian Blue analogue, electrochemical ion pumping, nanomaterials

## Abstract

The electrochemical ion pumping device is a promising alternative for the development of the industry of recovering metals from natural sources—such as seawater, geothermal water, well brine, or reverse osmosis brine—using electrochemical systems, which is considered a non-evaporative process. This technology is potentially used for metals like Li, Cu, Ca, Mg, Na, K, Sr, and others that are mostly obtained from natural brine sources through a combination of pumping, solar evaporation, and solvent extraction steps. As the future demand for metals for the electronic industry increases, new forms of marine mining processing alternatives are being implemented. Unfortunately, both land and marine mining, such as off-shore and deep sea types, have great potential for severe environmental disruption. In this context, a green alternative is the mixing entropy battery, which is a promising technique whereby the ions are captured from a saline natural source and released into a recovery solution with low ionic force using intercalation materials such as Prussian Blue Analogue (PBA) to store cations inside its crystal structure. This new technique, called “electrochemical ion pumping”, has been proposed for water desalination, lithium concentration, and blue energy recovery using the difference in salt concentration. The raw material for this technology is a saline solution containing ions of interest, such as seawater, natural brines, or industrial waste. In particular, six main ions of interest—Na^+^, K^+^, Mg^2+^, Ca^2+^, Cl^−^, and SO_4_^2−^—are found in seawater, and they constitute 99.5% of the world’s total dissolved salts. This manuscript provides relevant information about this new non-evaporative process for recovering metals from aqueous salty solutions using hexacianometals such as CuHCF, NiHCF, and CoHCF as electrodes, among others, for selective ion removal.

## 1. Introduction

The efficient supply of energy is the most important issue for multiple engineering industries and society. Energy availability and sustainability could be associated with different metal recovery technologies and the use of critical and valuable elements, including rare earth elements (REEs) [1], promoting scenarios to develop and put in practice an array of new technologies that allow minimizing both environmental impact and global warming. Additionally, to confront the high risk of future metal supply deficits, it is imperative to effectively integrate a set of sustainable recycling practices into the metal production scheme. In particular, for the case of Li, controversial debates have recently been generated related to its resource availability and future demand, especially to cover the growing electric market for cars and energy storage systems at an industrial level, which has led to variability and uncertainty in its price. Since 2012, the largest amount of lithium (approx. 83%) has originated from brines and natural saline waters, and its extraction produces a significant negative environmental impact. In processing facilities based on natural brines, lithium is concentrated through solar evaporation, which is a very slow process and the controlling stage to meet the growing demand for this mineral. In considering that, on the one hand, the cheapest form of Li for industrial use is lithium hydroxide (LiOH), and on the other hand, Li produced from natural brines is mainly in the form of lithium carbonate (Li_2_CO_3_), the expectation that brine-based lithium can be a strategic resource (similar to what copper is for Chile) is unfavorable in comparison with mining based on rock extraction. Lithium hydroxide comes from mining based on rock extraction, and in the case of brine processing, transforming Li_2_CO_3_ to LiOH requires an additional cost that eliminates the competitive advantages of producing it. Unfortunately, the current Li^+^ extraction method in Chile (the evaporative method) does not allow for direct production of Li^+^ without the intermediate LiOH compound.

The oceans, the highland brine of the Andes mountain range, geothermal waters, and hypersaline solutions from reverse osmosis plants contain a large number of dissolved ions that in principle could be extracted with other conventional, less complex, expensive, and polluting processes, compared to typical land mines [1]. Osmosis plants reject a huge amount of mineral salts that eventually end up in the sea as dissolved ions, and so the oceans can be considered as a huge reservoir of valuable metallic ions, including lithium ions, for the extraction of which is needed the use of sustainable and efficient processes. The recovery of valuable metal ions from natural saline and hypersaline solutions has been carried out successfully for solutions rich in Li^+^. According to the Mg^2+^/Li^+^ mass ratio, high-altitude brine in the Atacama salt flat can be divided into low-Mg^2+^/Li^+^, < 8, and high-Mg^2+^/Li^+^, > 8. Li^+^ harvesting is known to be easier in low-Mg^2+^/Li^+^ brine, due to its relatively high Li^+^ content. However, effective separation of Mg^2+^ and Li^+^ from high-Mg^2+^/Li^+^ brine is a more difficult and expensive process [2].

The use of electrochemical processes would allow for selective capture of cations or anions such as the Li^+^ ion and the Cl^−^ ion present in saline and hypersaline solutions directly, using non-conventional renewable energies such as solar energy, wind energy, geothermal energy, solar energy biomass, and/or blue energy for the energy source of the process, and using intercalation cathode materials such as Prussian Blue analogue (PBA). It is foreseeable that industrial companies adopting a well-developed and sustainable technology compatible with non-conventional renewable energy sources will not only be at the forefront of sustainable mining, but will also be an example of a step in the right direction. Electrochemical technology has the potential versatility to be adapted for recovering other ions with high commercial value besides Li, such as Cu. Such a possibility can be linked to the energy industry and energy storage industry devices whose direction of development will be a result of innovative technologies yet to come.

## 2. Theoretical Background of Electrochemical Ion Pumping

**Figure 1:**
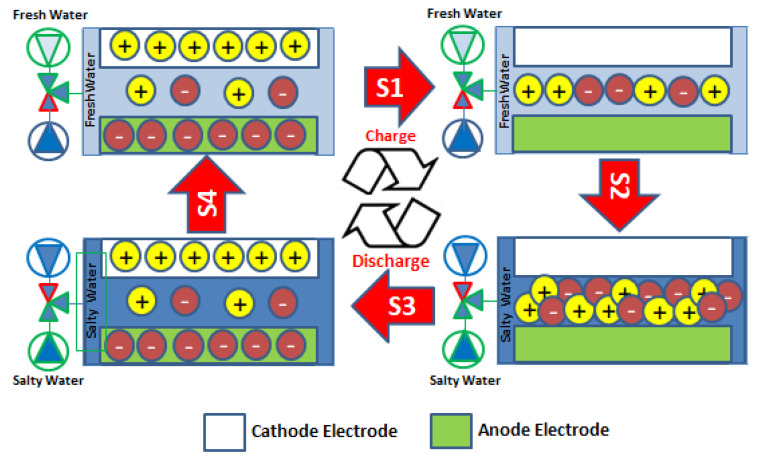
Schematic representation of the systematic work of the mixing entropy battery.

Kanoh et al. invented an electrochemical method to recover Li^+^ ions from seawater using a manganese oxide $\left(\lambda -MnO_{2}\right)$ on a Pt substrate as the working electrode, Pt wire as the counter electrode, and calomel as the reference electrode [2]. They also studied the adsorptive properties and the mechanism for Li^+^ insertion into the $\lambda -MnO_{2}$ structure in contact with solutions [3,4,5]. The investigations showed that, during Li^+^ insertion into the $MnO_{2}$ electrode, the Mn^4+^ is reduced to Mn^3^ [3,6]. The process is not spontaneous and requires control of the pH, and the energy consumption is around 33 W·h/mol. In this method, the HER, ORR, and OER mechanisms balance the electrochemical circuit during Li^+^ capturing and release, respectively [3,4,5]. The electrochemical ion pumping device is based on the previous mixing entropy battery (MEB) and desalination battery developed by Fabio La Mantia and Mauro Pasta. It consists of a reversible electrochemical system, where the Na^+^, Li^+^, and Cl^−^ ions present in the salty solution are stored in their respective electrodes [7,8]. The proposed MEB operates selectively, in which the anionic and cationic electrodes interact with Cl^−^ and Na^+^ ions in a multistep circuit according to Figure 1. Firstly, due to the low ionic strength of the solution, the battery is charged when the cations (M^+^) and anions (A^−^) are removed from the electrodes (step 1). After that, changes in the cell voltage can be observed due to the exchange of the recovery solution for the concentrated solution (step 2), generating an increase in the cell voltage known as Voltage Rise [4,5,7,8,9,10,11,12,13,14]. Furthermore, the battery is discharged with a high cell voltage, capturing M^+^ and A^−^ ions from the concentrated solution and interspersing those within the crystalline structure of the electrodes, generating energy (step 3). Finally, the concentrated solution is exchanged for a recovery solution with a low-ionic-strength solution (step 4), leaving the device ready to start a new cycle. Steps 2 and 4 contain no power consumption or generation. In the MEB device, a Cl^−^ ion-selective anionic electrode made of Polypyrrole (PPy) and a cationic electrode of $K\left[NiFe{\left(CN\right)}_{6}\right]$ which interacts selectively with the metallic ion M^+^ were used. The global and its decomposition into their partial reactions can be written with the reactions 1, 2, and 3, respectively:

(1) $$K\left[NiFe{\left(CN\right)}_{6}\right]+PPy+M^{+}+{Cl}^{-}↔MK\left[NiFe{\left(CN\right)}_{6}\right]+PPyCl$$

(2) $${K\left[NiFe{\left(CN\right)}_{6}\right]}_{\left(\alpha \right)}+M_{\left(\varepsilon \right)}^{+}+e^{-}↔{MK\left[{NiFe\left(CN\right)}_{6}\right]}_{\left(\beta \right)}$$

(3) $$PPy{Cl}_{\left(\beta^{’}\right)}+e^{-}↔{PPy}_{\left(\alpha^{’}\right)}+{Cl}_{\left(\varepsilon \right)}^{-}$$

where α is the $K\left[NiFe{\left(CN\right)}_{6}\right]$ phase, β is the $MK\left[NiFe{\left(CN\right)}_{6}\right]$ phase, ε is the electrolyte, $\beta^{\prime }$ is the PPyCl phase, and $\alpha^{\prime }$ is the PPy phase. The Nernst potential of the two half-cell reactions concerning the normal hydrogen electrode (NHE) is given by

(4) $$E_{+}=E_{+}^{0}+\frac{RT}{F}ln\left[\frac{a_{M,\varepsilon }}{a_{M,\beta }}\right]$$

(5) $$E_{-}=E_{-}^{0}-\frac{RT}{F}ln\left[a_{Cl,\varepsilon }\right]$$

where $E_{+}$ and $E_{-}$ are the equilibrium potential of the electrodes, $E_{+}^{0}$ and $E_{-}^{0}$ are the standard potential of the electrodes, $a_{M,\beta }$ is the activity of the metal in the solid phase, $a_{M,\varepsilon }$ is the activity of the metal ions in the electrolyte, $a_{Cl,\varepsilon }$ is the activity of chloride ions in the electrolyte, R is the ideal gas constant, T is the temperature, and F is the Faraday constant. Considering that the activity of the metal in the solid phase is equal to one, the cell potential (${\Delta E}_{eq}$) at equilibrium can be calculated according to the following equation:

(6) $${∆E}_{eq}={∆E}^{0}+\frac{RT}{F}ln\left[a_{M(\varepsilon )}\right]+\frac{RT}{F}ln\left[a_{Cl(\varepsilon )}\right]$$

where ${∆E}^{0}$ is the cell potential under standard conditions. In this mixing entropy battery, the electrical work performed by the cell, W, is given by the contribution of the thermodynamic and kinetic work and is expressed by the following equation:

(7) $$W=\int_{1}^{2}\Delta E_{eq}\cdot I\cdot dt+\int_{3}^{4}\Delta E_{eq}\cdot I\cdot dt+\int_{1}^{2}\eta \cdot I\cdot dt+\int_{3}^{4}\eta \cdot I\cdot dt=W_{th}+W_{k}$$

where η is the overpotential associated with charge and discharge, I is the current intensity, t is the time, and $W_{th}$ and $W_{k}$ are the electrical work from the thermodynamic and kinetic contributions, respectively. Kinetic effects are usually related to overpotentials and are essential in determining the amount of energy extracted. However, kinetic limitations come hand in hand with mass diffusion processes. Equation (7) is related to the electrical work or energy consumed and is given by the integral along the cycle of cell potential (∆E) concerning the charge (q). The Gibbs free energy variation, which determines the potential ∆E, is only due to entropic factors, the final and initial concentration of the ions in the brine, and the recovery solution [4].

In the case of the Li^+^ capture process, Equation (7) should be normalized concerning the amount of Li+ recovery as an ion of interest. In this method, the process is thermodynamically favored to generate energy, transforming the chemical energy liberated in the mixed solutions with different concentrations of salt, and consequently, electrical energy is produced. This electrochemical cell is called salinity gradient energy or blue energy [10,11], and works in a similar form to a galvanic cell. However, the use of brine solutions in the battery device shows low energy consumption, and it is necessary to expend energy to transfer Li^+^ to the recovered solution in the cycle shown in Figure 1. This discrepancy, concerning a cell favored by thermodynamics, is mainly due to the loss of energy through ohmic drop, concentration overvoltage, water splitting, and oxygen reduction.

Analysis of energy dissipation shows that, in a mixing solution system, the entropy energy dissipation is estimated at 2.2 kJ per liter of fresh water when river water enters the sea [7,9,12,13,14,15,16,17]. Under these considerations, the energy produced in a salinity gradient system like river water and seawater could potentially reach up to 2 TW, which is more than 13% of the worldwide energy consumption [7,12,18,19,20,21,22]. The theoretical non-expansion work that can be produced from mixing a relatively concentrated salt solution h (brine) and a dilute salt solution l (recovery solution) at constant pressure p and absolute temperature T to give a brackish solution m is defined by the Gibbs energy of mixing ∆G_mix_ [7,23]:

(8) $$∆G_{mix}=∆G_{m}-\left({∆G}_{h}+{∆G}_{l}\right)$$

Considering that solutions are ideally dilute and no heat is lost or gained (i.e., ${∆H}_{mix}=0$) [23,24], the Gibbs energy can be determined from changes in molar entropy with the following equation:

(9) $${∆G}_{mix}=-\left(n_{h}+n_{l}\right)T{∆S}_{mix,m}-({-n}_{h}T{∆S}_{mix,h}-n_{l}T{∆S}_{mix,l})$$

where ${∆S}_{mix}$ is the mixing entropy and n is the number of moles. Under isothermal conditions, the Gibbs free energy related to the transfer of species i from system 1 to system 2 (${∆G}_{i1,2}$) can be determined by an additive contribution of both systems separately, according to the following equations:

(10) $${∆G}_{\begin{array}{c} i1,2 \end{array}}={∆G}_{i,1}+{∆G}_{i,2}=G_{{i,1}_{F}}-G_{{i,1}_{I}}+G_{{i,2}_{F}}-G_{{i,2}_{I}}$$

(11) $$\frac{{∆G}_{\begin{array}{c} i1,2 \end{array}}}{RT}=n_{i,1_{F}}ln\left(C_{i,1_{F}}\right)-n_{i,1_{I}}ln\left(C_{i,1_{I}}\right)+n_{i,2_{F}}ln\left(C_{i,2_{F}}\right)-n_{i,2_{I}}ln\left(C_{i,2_{I}}\right)$$

where “F” and “I” denote the final and initial conditions of each system, respectively. Therefore, applying the Nernst equation for the global reaction in Equation (1) and considering the lithium ion case, the equilibrium potential between the two electrodes can be written as follows [25]:

(12) $$E_{Cell}=E_{Cell}^{0}+2\frac{RT}{F}ln\left[C_{LiCl}\right]+2\frac{RT}{F}ln\left[\gamma_{LiCl}\right]$$

where $E_{Cell}^{0}$ is the standard cell voltage, $C_{LiCl}$ is the LiCl concentration, and $\gamma_{LiCl}$ is the mean activity coefficient of LiCl. The dependence of $\gamma_{LiCl}$ on $C_{LiCl}$ is described by the extended Debye–Hückel equation that considers only Cl^−^ and Li^+^ ions in solution, which is as follows:

(13) $$ln\left[\gamma_{LiCl}\right]=-\frac{A\sqrt{C_{LiCl}}}{1+B\sqrt{C_{LiCl}}}$$

where A and B are constants that depend on the temperature and solvent properties.

## 3. Metal Recovery Parameters

To date, many scientific articles have been published showing that the recovery of metals from aqueous solutions using intercalation electrodes that operate under pseudocapacitive processes is feasible. A main operating constraint for the recovery process is that the intercalation can not be specific for one cation, but is in tandem with other ions. For this reason, many parameters, which under certain operating conditions are difficult to compare, have been proposed to characterize the efficiency of metal recovery. Considering the popularity criterion, only some of these parameters have been selected for review and evaluation in the recovery process.

For Li^+^ recovery, the selectivity during intercalation originates from the interference of other co-existing cations, referred to as co-cations. Equation (14) represents how much Li^+^ could be incorporated into the electrode material from the salty solution without co-intercalating other cations [26,27].

(14) $$\alpha_{M}^{Li}=\frac{C_{Li}}{C_{M}}$$

where $C_{Li}$ is the Li concentration and $C_{M}$ is the concentration of a secondary cation such as Na^+^, K^+^, Ca^2+^, or Mg^2+^ after one full recovery cycle in the recovery solution. It is directly dependent on the concentration of co-cations in the recovery solutions and indirectly dependent on the concentration of ions in the salty solution. Higher concentrations of co-cations would cause an increase in the co-intercalation of other ions and a decrease in the value of $\alpha_{M}^{Li}$ [27,28,29]. The selectivity of Li^+^ has also been calculated with the following expression:

(15) $$\alpha_{M}^{Li}=\frac{K_{D}^{Li}}{K_{D}^{M}}$$

where $K_{D}^{Li}$ is the distribution coefficient of Li^+^ and $K_{D}^{M}$ is the distribution coefficient of other cations in transit from the brine solution to the electrode material. The distribution coefficient ($K_{D}$) applied for ions other than Li^+^ is expressed in terms of the insertion capacity ($Q_{f}$) in the Li-capturing electrode to the final concentration in the brine solution [27,29,30].

(16) $$K_{D}=\frac{Q_{f}}{C_{f}}$$

(17) $$Q_{f}=\frac{\left(C_{0}-C_{f}\right)}{m}\cdot V_{f}$$

where $Q_{f}$ is calculated from the initial concentration ($C_{0}$) and final concentration ($C_{f}$) of the cation in the brine solution with a known volume ($V_{f}$) and mass material (m).

Another important parameter for evaluating the efficiency of metal recovery related to the purity of the Li^+^ in the recovery solution is the total selectivity coefficient ($A_{Li})$, expressed in the following equation [27,28]:

(18) $$A_{Li}=\frac{C_{Li}}{\sum C_{M}^{t}}$$

where $C_{M}^{t}$ is the concentration of any cation in solution, including Li^+^.

Using a principle based on the separation coefficient, another parameter is proposed. This is the separation coefficient (SF) of Li^+^ relative to a co-cation and is calculated from their molar concentration ratio at two instances [27,31].

(19) $$SF={\left(\frac{C_{Li}}{C_{M_{e}^{+}}}\right)}_{t}/{\left(\frac{{C\prime }_{Li}}{{C\prime }_{M_{e}^{+}}}\right)}_{0}$$

where $C_{Li}$ and $C_{M_{e}^{+}}$ are the concentrations of Li^+^ and ${Me}^{n+}$ in the recovery solution at instance t, and ${C^{\prime }}_{Li}$ and ${C^{\prime }}_{M_{e}^{+}}$ are the concentrations in the brine solution at instance 0.

This factor represents the Li^+^ purity in the brine (expressed as subscript 0) and in the recovery solution (expressed as subscript t), and visually indicates how many times the lithium purity changes between 0 and t.

## 4. Energy Efficiency Consumption

The coulombic efficiency is a parameter indicating the percentage of the current applied that is effectively used to extract Li^+^, and is defined as [28,29].

(20) $$\eta_{Li}=\frac{FC_{Li}V}{Q}\times 100$$

where F is the Faraday constant, V is the volume of the recovery cell, and Q is the total charge flow during the Li^+^ extraction step. The efficiency is especially affected by two parameters: firstly, from the selectivity in capturing the ion of interest (an electrode that intercalates a large amount of unwanted cation or provides secondary reactions would display a low coulombic efficiency); secondly, the intrinsic reversibility of the Li ion intercalation reaction.

## 5. Adsorption Parameters

For the electrode processes that take place in the MEB, several parametric representations of adsorption models are based on different isotherm adsorption models. Some expressions are given below [32,33].

Langmuir isotherm model

(21) $$\frac{C_{e}}{Q_{e}}=\frac{1}{Q_{m}\cdot K_{L}}+\frac{C_{e}}{Q_{m}}$$

(22) $$R_{L}=\frac{1}{1+K_{L}C_{0}}$$

Freundlich isotherm model

(23) $$lnQ_{e}=lnK_{F}+\frac{lnC_{e}}{n}$$

Temkin isotherm model

(24) $$Q_{e}=\frac{R\cdot T}{b_{T}}ln\left(a_{T}\right)+\frac{R\cdot T}{b_{T}}ln\left(C_{e}\right)$$

Dubinin–Radushkevic isotherm model

(25) $$ln\left(Q_{e}\right)=ln\left(Q_{m}\right)-K_{DR}\cdot \varepsilon^{2}$$

(26) $$\varepsilon =R\cdot T\cdot ln\left(1+\frac{1}{C_{e}}\right)$$

Redlich–Peterson isotherm model

(27) $$Q_{e}=\frac{K_{RP}\cdot C_{e}}{1+\alpha_{RP}\cdot C_{e}^{\beta RP}}$$

where $C_{e}$ is the equilibrium concentration, $C_{0}$ is the initial concentration, $Q_{e}$ is the adsorption capacity, $Q_{m}$ is the theoretical maximum adsorption capacity, $K_{F}$ is a constant indicative of the relative adsorption capacity of the adsorbent, $K_{L}$ is the adsorption isotherm constant, R is the ideal gas constant, T is the temperature, and $b_{T}$ is the adsorption heat of the Temkin isotherm. The dimensionless constant $R_{L}$ for the Langmuir model indicates either (i) $R_{L}=0$: the adsorption is irreversible; (ii) $0<R_{L}<1$: favorable condition; (iii) $R_{L}=1$: linear condition; and (iv) $R_{L}>1$: unfavorable condition.

$K_{DR}$ is the activity coefficient of the Dubinin–Radushkevich isotherm, ε is the Polanyi potential. $\beta_{RP}$ is the exponent from the Redlich–Peterson model between 0 and 1.

## 6. Compilation of Metal Hexacyanoferrate Synthesis Methods

Although Prussian Blue analogues are promising catalysts, PBs are generally prepared on the nanoscale from reactions of metal ions with hexacyanometalates in solution [34,35], making them less practical, especially for solution-based reactions. Although some attempts have developed substrate-supported PBs, many of them are still very small and the PBs are not grown homogeneously. However, there is still an urgent demand to develop substrate-supported PB that is catalytically efficient and easy to prepare, use, and recover. Different methods for obtaining Prussian Blue used by different researchers are discussed below.

Doumic et al. immobilized nanoparticles of Prussian Blue over granular activated carbon (GAC) to improve the recovery of PB by the Fenton reaction. While PB grew on the GAC surface, the resulting PB coverage on GAC was not uniform due to irregular GAC surface morphology. The GAC pores were also blocked by the impregnated PB, losing the advantageous properties of the GAC supports. Furthermore, the target compounds adsorbed on the GAC instead of participating in catalytic reactions [36]. A previous study also used cellulose fibers to support PB; however, the resulting fiber-supported PB was still very fine (nanoscale), making it less practical. Prussian Blue nanoparticles are powerful adsorbents for the selective elimination of radioactivity [37]. Chen et al. prepared FeCo alloy nanoparticles coated with Nitrogen-doped graphene layers via carbonization of Fe-Co PBA. The derived N_2_-doped graphene layers coated on FeCo alloy nanoparticles exhibited an onset overpotential of 88 mV for HER and required a 262 mV overpotential to obtain the current density of 10 mA cm^−2^ in 0.5 M H_2_SO_4_ [38]. Yamauchi et al. synthesized a series of PBAs including Co-Fe PBA, Ni-Fe PBA, Ni-Co PBA, and Ni-Cr PBA by mixing hexacyanometal complex ions with metal ions in water. In a typical procedure, metal salt (nickel chloride or cobalt chloride) and sodium citrate were dissolved in water to form a clear solution A. K_3_[M(CN)_6_] (M = Fe, Co, Cr) was dissolved in water to form a clear solution B. A and B were mixed with magnetic stirring until the mixture became clear. The obtained solution was aged for 24 h. The PBAs as precipitates were collected using centrifugation [34]. Zhang et al. fabricated PBA nanocubes using metal hydroxides/oxides as precursors and templates. A series of PBA nanocubes could be fabricated on cobalt oxide, manganese oxide, copper hydroxide, cobalt fluoride hydroxide, nickel-cobalt hydroxide monometallic or bimetallic nanosheets by etching these metal hydroxides/oxides with K_3_[M(CN)_6_] (M = Co, Fe) [39]. Lou et al. reported an anion exchange/etching strategy to convert Ni-Co PBA nanocubes into nickel sulfide nanostructures. In a typical procedure, Ni-Co PBA nanocubes were dispersed in ethanol with the aid of ultrasound to obtain a homogeneous suspension. Subsequently, the Na_2_S aqueous solution was added to the Ni-Co PBA nanocube suspension with continuous stirring. The resulting mixture was transferred to a Teflon-lined stainless steel autoclave and held at 100 °C for 6 h in an electric oven [40]. Guo et al. synthesized N-doped carbon nanocages of core and shell NiFe alloy through carbonization of Ni-Fe PBA at 600 °C for 3 h in an H_2_/Ar atmosphere. The Ni-Fe PBA nanocube precursor produced a NiFe alloy and ensured the in situ generation of N-doped carbon without additional sources of N and C. During the heat treatment process, Ni^2+^ and Fe^3+^ on the Ni-Fe PBA surface were reduced with hydrogen to generate a NiFe alloy. Pyrolysis of the cyano ligand in situ generated nitrogen-doped graphite carbon and was coated with a NiFe alloy to obtain a core and shell material [41]. Guo et al. successfully converted Co-Fe PBA nanocubes into hollow porous CoFe_2_O_4_ nanocubes, which was prepared by heating the Co-Fe PBA precursor as prepared at 350 °C with a heating rate of 1 °C/min under flow of air, and then kept at 350 °C for 4 h. They found that the CoFe_2_O_4_ nanocubes had a hollow rather than a solid structure, which could be attributed to rapid mass transport through the layers during the annealing process [42].

A new procedure for preparing monodisperse Prussian Blue nanoparticles was investigated. Using gelatin as a protective colloid, a solution of citric acid was added to the initial solution containing Fe(NO_3_)_3_, K_3_Fe(CN)_6_, and HNO_3_, where the acid was added to prevent coagulation by crosslinking between the gelatin molecules. by F_e_(CN)_6_^3−^ ions. Employing a Fe reduction reaction, monodisperse Prussian Blue nanoparticles with an average size of 70 nm were obtained by using citric acid at 35 °C [43].

Table 1 shows the advantages and disadvantages of the different methods of obtaining Prussian Blue, with their respective preparation methods.

To meet these requirements, macroscale materials appear as the most attractive supports for PB because such materials can be easily handled in industrial operations and can be conveniently separated from solutions by gravity. Macroscale materials can also be packed in columns for continuous reactions. However, suitable macroscale supports must be robust, chemically stable, industrially available, and inexpensive. Most importantly, these macroscale scaffolds must be functionalized at the surface to bind PB precursors to grow through layer-by-layer self-assembly [44].

Ion exchange (IE) resins are used as macroscale supports to grow PB NPs. IE resins are robust, inexpensive, and stable, allowing them to be widely used in solution-based processes, especially aqueous reactions. The inherent surface charges of IE resins make them readily available for binding with PB precursors through electrostatic attractions. The spherical shapes of the resins can also allow PB to grow homogeneously on the outer surfaces, maximizing physical contact between the PB and target compounds [45]. These PB precursor-functionalized resins have been prepared for ion exchange applications or adsorption.

### 6.1. Obtaining Method of Prussian Blue over Resin (PB_R)

**Figure 2:**
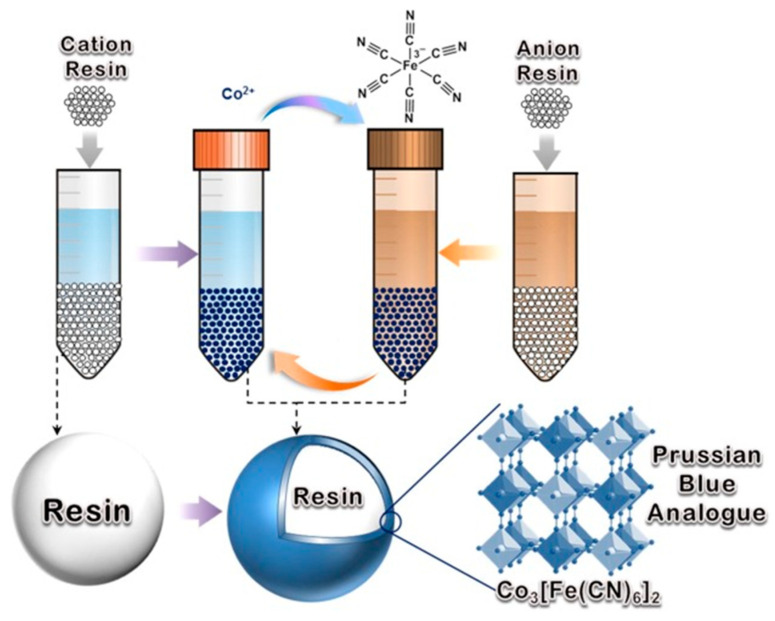
Scheme of preparation of macrosphere-supported nanoscale Prussian Blue analogues (PB_R) by self-assembly.

To prepare PB_R using cathodic resin (CR), the typical procedure starts by adding 1 g of CR to 40 mL of an aqueous solution containing 0.435 g of Co(NO_3_)_2_·6H_2_O and 0.2 g of sodium citrate in a tube for centrifugation. The tube is then placed on an orbital shaker to equilibrate CR with Co^2+^ at room temperature for 2 h. Subsequently, the cobalt-deposited CR is separated from the cobalt solution by precipitation, washed with deionized water, and then added to another centrifuge tube containing 0.33 g K_3_Fe(CN)_6_ and 0.2 g citrate of sodium in 40 mL of deionized water. The mixture is then stirred at room temperature for another 2 h. The aforementioned procedure is repeated for two additional cycles to grow PB NPs on the CR surface. The resulting product is thoroughly rinsed with deionized water and finally dried at 338 °K overnight to yield PB_CR. The procedure to prepare PB_AR is similar to that for PB_CR, except that AR is first added to 40 mL of K_3_Fe(CN)_6_ solution and then to the aqueous Co^2+^ solution to complete a self-assembly cycle. The scheme for the preparation of PB_CR and PB_AR is illustrated below in Figure 2 [45].

### 6.2. Prussian Blue on an Ordered Mesoporous Carbon (OMC) Substrate

Ordered mesoporous carbons are of great interest for the fabrication of new classes of advanced carbons. The ability of OMCs to promote the electron transfer reactions of important molecules, such as l-cysteine, dopamine, and epinephrine, has made them attractive for the construction of various electrochemical sensors.

**Figure 3:**
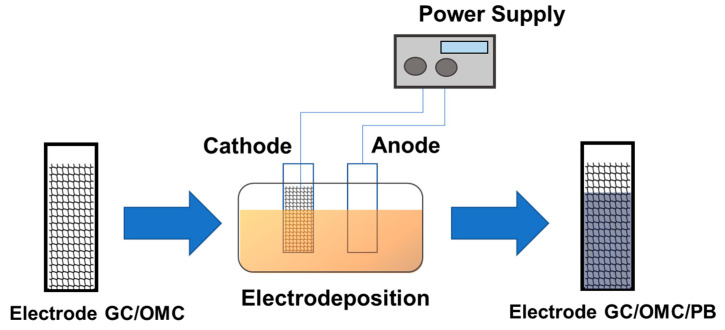
Scheme for obtaining Prussian Blue supported on a GC/OMC electrode.

Jing Bai et al. [46] used the electrodeposition method, using a modified glassy carbon (GC) electrode previously prepared with ordered mesoporous carbons (OMC) to deposit the Prussian Blue on top show in Figure 3. The first step is the treatment of the modified glassy carbon with ultrasound using ethanol and double-distilled water. Subsequently, the authors dispersed 5 mg of OMC in 10 mL of N, N-dimethylformamide (DMF) with the help of ultrasonic oscillation to give a 0.5 mg/mL black suspension, placing only 3 μL of the suspension in the surface of the modified glassy carbon electrode, and with the help of an infrared lamp the solvent used was allowed to dry. Electroplating of Prussian Blue was performed as follows: the OMC-treated GC electrode was immersed in an electrochemical cell with an unstirred solution of 2 mM FeCl_3_·6H_2_O, 2 mM K 3Fe(CN)_6_, 0.1 M KCl, and 0.1 M HCl, applying a constant potential of +0.4 V for 200 s. Then, the appliance was washed with water and transferred to a solution containing 0.1 M KCl + 0.1 M HCl and activated with electrochemical cycling between +0.4 V and −0.5 V (20 cycles) at a sweep rate of 50 mV/s. Finally, the appliance was washed with double-distilled water and dried in ambient air.

## 7. Salty Electrolytes as Sources of Metals

### 7.1. Seawater

The water resources on the planet are principally distributed in seawater which covers nearly 97% of the Earth, with the other 3% covered by fresh water resources (groundwater, rivers, lakes, among others) and water in ice caps and glaciers. Seawater is a concentrated saline solution that contains cations, such as sodium and magnesium, and anions, such as chloride and sulfate, as major dissolved ions. In addition to other dissolved ions (Ca^2+^, K^+^, HCO_3_^−^, Br^−^, Sr^2+^, Li^+^, B, and F^−^, among others), the standard seawater has a salinity in a range of 33 to 37 g/L, corresponding to 5 × 10^16^ tons of total mass [47,48,49,50]. The concentration of ions in seawater depends mainly on two factors: their crustal abundance and the existence of water-soluble species. These two constraints account for the spread in the concentration values. The interest in seawater in terms of water and metal resources is highly understandable if we compare the amount of metal ions dissolved in marine waters with the total mass of terrestrial minerals extracted in the world and the volume of water processed. It has estimated that the mass of seawater that must be processed to obtain certain elements varies from 1.4 × 10^11^ tons for lithium up to 1.58 × 10^17^ tons for cobalt [48]. Many researchers studied the harvesting of a variety of toxic metals (e.g., Cd, Hg, Pb, and Zn), radionuclides (e.g., Ce, Cs, Sr, and U), precious metals (e.g., Ag, Au, Pd, and Pt), and light metals (e.g., Mg and Al) using different techniques and processes. On the other hand, multivalent cations and anions such as Na^+^, K^+^, Mg^2+^, Ca^2+^, SO_4_^2−^, Cl^−^, and HCO_3_^−^, and traces of elements such as B^+^, Li^+^, and Cu^2+^ are present in seawater, which also can be recovered. However, elements such as Co^2+^, Cr^3+^, Fe^2+^, Mn^2+^, Sr^2+^, Pb^2+^, and Zn^2+^ have not yet been well documented nor economically considered within metals that can be recovered from aqueous solutions or natural saline waters [51]. Under these considerations, the use of electrochemical cells with intercalation of polyvalent cations, such as Mg^2+^, Ca^2+^, Zn^2+^, Al^3+^, or Y^3+^, opens up the possibility of developing batteries with potential practical applications [52]. Many metallic and non-metallic elements are dissolved in low concentrations in seawater and could be recovered in significant proportions [53]. These are metallic cations such as Na^+^ (1.4 × 10^16^ ton), K^+^ (5.1 × 10^14^ ton), Li^+^ (2.31 × 10^11^ ton), Mg^2+^ (1.68 × 10^15^ ton), Ca^2+^ (5.34 × 10^14^ ton), and Zn^2+^ (6.5 × 10^9^ ton), whose combined amount represent about 77% of the total dissolved salts [48]. However, seawater contains other ions of interest, such as Cu^2+^ (1.17 × 10^9^ ton), Ni^2+^ (8.58 × 10^9^ ton), Au (1.43 × 10^7^ ton), Co^2+^ (5.07 × 10^8^ ton), and U (4.29 × 10^9^ ton) [48]. One of the great disadvantages of recovering Li^+^ from the sea is that it is highly diluted, which makes this natural source an unviable alternative due to the high costs involved in recovering this metal from the seawater.

### 7.2. Brine from Reverse Osmosis Plants

Fresh water has become an important topic that is crucial to improve the quality of life of the global population and its growth rate. Unfortunately, the availability of adequate-quality drinking water is affected by human activities. Increasing amounts of fresh water will be required in the years to come as a result of population growth, improved quality of life, and expansion of industrial and agricultural activities. At the present rate of population growth, humans will consume 70% of available freshwater by 2025, which will increase up to 90% within 25 years, at which point the world population living in water-scarce areas is expected to increase to 3.9 billion. In this context, the desalination of seawater or brackish water is becoming the most feasible source of obtaining fresh water. Seawater desalination processes require electrical or thermal energy to separate the seawater into two streams: a freshwater stream containing a low concentration of dissolved salts and a concentrated brine stream. A wide variety of desalination technologies have been developed over the years. The use of brine from seawater desalination plants is an interesting alternative to consider since it is currently discharged in large quantities into the sea. The reverse osmosis brine has a salt concentration of up to three times the salt concentration of seawater, a process that is not without its problems. On the one hand, there is a great requirement for electrical energy for the operation of these plants, which generates carbon dioxide and increases the greenhouse effect [51]. Furthermore, the environmental impact caused by the generation of brine that derives from this process directly affects the marine ecosystem [54]. This brine, being denser than seawater, can be deposited in the same place where it is discharged, and thus disturb the local ecology, depending on the physical conditions of the area, namely, sea currents and waves, affecting living conditions and the development of the species that live in it. Another interesting aspect is that when a solution has a high salinity, the concentration of dissolved O_2_ tends to drop, generating problems for the coastal ecosystem. These reverse osmosis systems require a large input of electrical energy, which represents 44% of the cost of desalination and is based on the use of selective membranes that are prone to fouling and require frequent replacement.

### 7.3. Altitude Brine

Currently, Chile is positioned as the country with the largest lithium reserves worldwide, which makes it a great competitor to countries such as Argentina, Bolivia, and Australia. It is estimated that Chile has lithium reserves equivalent to 7.5 million tons, which is 52% of the lithium available globally. Lithium extraction from the Salar de Atacama is carried out by pumping large quantities of brine (20,000 m^3^/d) from underground reservoirs, and then storing it in large shallow ponds [4]. There, the brine is concentrated to a concentration of 6 g/L of Li^+^ for 12 to 24 months, losing large amounts of water (>95%) through solar evaporation, estimated at 10,000 m^3^ of water/ton of Li_2_CO_3_ produced. Unfortunately, this process generates an irreversible environmental impact in the hydrographic basin of the Atacama salt flat. Preliminary studies related to the environmental impact of lithium mining indicate that the increasing and constant extraction of lithium through evaporative methods could irreversibly compromise the ecosystem of the salt flat and deplete the water reserves in the area. The current lithium extraction process from high-altitude brine is carried out via evaporation for between 12 and 24 months until the LiCl concentration rises to 6 g/L and the more abundant salts start to precipitate, such as as halite (NaCl), potassium carnalite $\left(KCl\cdot Mg{Cl}_{2}\cdot 6H_{2}O\right)$, sylvinite (NaCl·KCl), lithium carnalite $\left(LiCl\cdot Mg{Cl}_{2}\cdot 6H_{2}O\right)$, gypsum $\left(CaSO_{4}\right)$, and bischofite $\left(Mg{Cl}_{2}\cdot 6H_{2}O\right)$ [4,55]. In addition to requiring long process times, the solar evaporative method only recovers around 70% of lithium present in the original brine, which also can drop up to 50% or less in the presence of high concentrations of magnesium $\left({Mg}^{2+}\right)$ and sulfate $\left({SO}_{4}^{2-}\right)$. Currently, this method is the most popular process for recovering lithium in South America. The options for lithium recovery from seawater or brine that have been tested are similar, such as (i) chromatography [56], (ii) ion exchange [57,58], (iii) liquid–liquid extraction [59], (iv) electrodialysis using ionic liquid membrane [60], and (v) co-precipitation [61]. Based on the above, it can be concluded that it is imperative to change the lithium extraction method to a more efficient way that avoids the loss of millions of cubic meters of water and is carbon neutral.

### 7.4. Geothermal Water

The geothermal water and deposits vary significantly in composition, and have an ionic distribution generated from natural reactions between underground flowing water through bed rocks or cooling magma. This kind of hot magmatic brine is encountered primarily in sediment basins and exhibits a considerable concentration of valuable metal ions. The geothermal water of El Tatio (Chile) provides ionic elements such as Li^+^ (44 mg/kg), Na^+^ (4800 mg/kg), K^+^ (800 mg/kg), Cs^+^ (17 mg/kg), Mg^2+^ (0.7 mg/kg), B^+^ (206 mg/kg), and Cl^−^ (9000 mg/kg) as major components, and contains minor and trace elements of Al, As, Ba, Cd, Cu, Fe, Mn, Ni, P, Pb, Rb, Si, Sr, V, and Zn. As such, it is a promising source for mineral recovery [59,62,63]. Different technologies like electrochemical switched ion exchange, selective precipitation, solvent extraction, electrochemical ion pumping, or adsorption are used for lithium enrichment, reaching efficiencies of 80%. However, it is relevant to indicate that the recovery of lithium also depends on the presence of total dissolved solids (TDS), which varies with increasing depth and temperature of geothermal fluid. In geothermal waters with temperatures above 150 °C, TDS concentration has been reported between 2.5 and 81 g/L, while in geothermal waters with temperatures in the range of 90–150 °C, TDS varies between 1.1 and 8.2 g/L.

### 7.5. Groundwater

Groundwater formed from hydrothermal systems close to copper deposits in the Atacama Desert are compositionally variable, with surface groundwater flows characterized by relatively low salinities (900–10,000 mg/L). The increase in salinity based on Cl^−^, Br^−^, Li^+^, and Na^+^ and conservative trace elements together with the relationship between O_2_ and H_2_ isotopes suggests that, in addition to water–rock reactions within the deposits, most of the compositional variation can be explained by groundwater mixing. The main large Chilean deposits contain Cl^−^ (11,205 to 80,467 ppm), Br^−^ (15.3 to 78.6 ppm), Ca^2+^ (730 to 4018 ppm), Mg^2+^ (300.4 to 1952.2 ppm), Na^+^ (9461 to 52,974.1 ppm), K^+^ (115.2 to 718.3 ppm), and Li^+^ (7.327 to 10.056 ppm) as major solutes, and Fe^2+^ (10.34 ppm), Mn^2+^ (0.563 ppm), Cs^+^ (0.185 ppm), Rb^+^ (0.364 ppm), and Sr^2+^ (8.858 to 35.944 ppm) as minor components. This composition suggests an attractive source of recovery metals for this arid zone [64].

## 8. Prussian Blue Analogue Electrode for Dissolved Metal Recovery

It is widely known that transition metal hexacyanoferrates are a significant family of polynuclear inorganic mixed-valence materials due to their interesting properties such as electrocatalysis [65], electrochromic [66], ion exchange selectivity [67], sense [68], and magnetism [69]. Recently, the Prussian Blue Analogue (PBA) has demonstrated the reversible insertion and extraction of mono- and multi-valent ions from aqueous solutions. The PBAs are valid candidates to replace the sodium-capturing electrode and spinel materials used in rechargeable batteries due to their low cost and excellent performance. These materials have an open crystal structure formed by a face-center cubic location of transition metal cations octahedrally coordinated to hexacyanometallate groups. The interstitial “A sites” within the structure can incorporate water molecules and large-sized ions. The general formula is $A_{x}P{\left[R{\left(CN\right)}_{6}\right]}_{1-y}\cdot {⌘}_{y}\cdot nH_{2}O$, where A is an alkali cation such as Ca^2+^, Mg^2+^, or H_2_O, P is the N-coordinated transition metal cation (P = Ni, Cu, Fe, Mn, Co, and Zn), $R{\left(CN\right)}_{6}$ is the hexacyanometallate anion, and ⌘ represents a hexacyanometallate vacancy. Both the N-coordinated and C-coordinated transition metals can be electrochemically active in this structure. Some studies have verified intercalation of Na^+^, Li^+^, Mg^2+^, Cu^2+^, Al^3+^, and NH_4_^+^ in PBA [13,17,70,71,72]. Ions introduced into PBA likely remain at least incompletely hydrated, and larger hydrated ions may diffuse through open channels created by these ferricyanide vacancies. The A sites have a 4.6 Å diameter and around 100 channels of 3.2 Å diameter, allowing for the rapid and reversible insertion of a variety of ions [73]. Meanwhile, their hydration cations’ radii are in the order of Cs^+^ (3.25 Å), Rb^+^ (3.29 Å), K^+^ (3.3 Å), NH4^+^ (3.3 Å), Na^+^ (3.6 Å), Ca^2+^ (4.1 Å), and Mg^2+^ (4.25 Å) [74,75].

The principal cathode PBA-based materials tested for metal and energy recovery from salinity gradient for to capture of Na^+^ ions from seawater are cobalt hexacyanoferrate (CoHCF) [25], nickel hexacyanoferrate (NiHCF) [76,77], and copper hexacyanoferrate (CuHCF) [13,17,19,22,24]. The use of the PBA material family in the MEB is the cheapest cathode material for blue energy and metal recovery [78]. The electrochemical reaction of PBAs with monovalent ions can be described according to the following formula [79]:

(28) $$P^{III}R^{III}{\left(CN\right)}_{6}\cdot nH_{2}O+2A^{+}+2e^{-}↔A_{2}P^{II}R^{II}{\left(CN\right)}_{6}\cdot nH_{2}O$$

**Figure 4:**
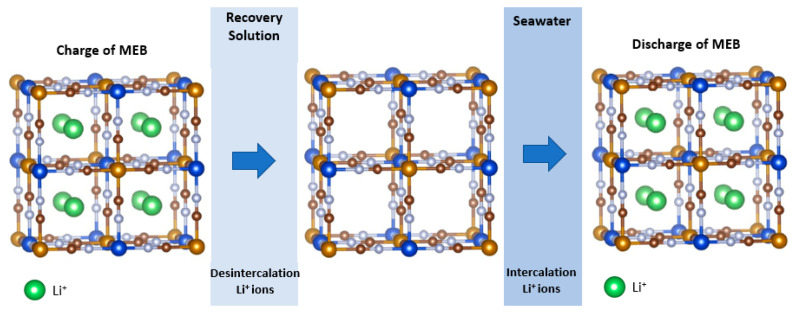
Schematic representation of recovering metals from natural saline solutions using PBA as a cathode electrode, charge of MEB (recovery metals), and discharge of MEB (captation metals from a natural source).

The particle size of these electrodes, ionic conductivity, the presence of zeolitic water, and the open structure helped to obtain high retention capacities, good kinetics, and high coulombic efficiency, indicating the versatile nature of these materials for unique applications such as the recovery of valuable metals from PLS, mining tailings, and/or for the recycling of battery electrodes, whether primary or secondary. Both materials remained stable in the intercalation of multivalent ions for up to 2000 cycles, but it was visualized that the retention capacities were decreasing when intercalated trivalent ions were compared with the divalent ions [70]. So far, only the aforementioned ions have been electrochemically recovered using battery materials from aqueous solutions. Figure 4 shows the intercalation and deintercalation of ions during the MEB process.

In general, the materials employed for intercalating dissolved metals exhibit a high degree of specificity, designed to accommodate guest atoms within their crystalline structures while being correlated with the dimensions of the unit cell. PBAs, for instance, have been characterized through X-ray diffraction (XRD) analysis, which confirms their classification within the cubic space group Fm$\overset{-}{3}$m. The dimension of the unit cell in PBA, as calculated with X-ray diffraction (XRD) analysis, was found to be 9.9954 Å. The unit cells of PBA consist of four units of molecules. Within these unit cells, Fe^2+^ ions representing R are situated at the corner and center positions of the cell faces. Simultaneously, the (CN)_6_^−^ group is positioned between R and Fe ions to establish the host structure’s network. The (CN)_6_^−^ group is oriented in a manner that facilitates the formation of R-C and R-N bonds. In the case of $A_{x}$ ions, out of the eight cations, only half of them can undergo reversible intercalation during electrochemical reduction, specifically the four during Fe^3+^ to Fe^2+^ redox centers. Likewise, during the oxidation of Fe^2+^ to Fe^3+^, deintercalation of the itinerant cation takes place. The Ax in PBA plays a crucial role in determining the dimensions of the crystalline structure. Depending on these dimensions, the structure can expand or contract to accommodate the specific ion of interest. PBA exhibits the ability to intercalate various cations with differing ease, a characteristic influenced by factors such as the diffusion coefficient and ionic radius of the specific ions. The selectivity of PBAs primarily stems from the intermolecular interstices within their crystalline structure, creating favorable spaces for atomic intercalation. These interstices are determined by the size of the target ion to be intercalated, along with its electro-affinity, which contributes to the overall intercalation selectivity of the PBA material. When considering cations, the absence of one or more electrons diminishes the repulsive force between the remaining electrons, leading them to draw closer to both each other and the positively charged nucleus. Consequently, cations tend to possess ionic radii smaller than their atomic radii. Conversely, anions exhibit the opposite behavior. The surplus of negative charge compels the electrons to spread apart, aiming to restore equilibrium to the electrical forces. This results in an ionic radius that exceeds the corresponding atomic radius. Several factors contribute to the selectivity of PBA materials, particularly toward metallic elements and cations. Additionally, the charge and discharge capacity of different PBAs are influenced by the ionic radius of the ions involved. Larger cations, such as Rb^+^ and Cs^+^, face challenges in entering the crystal structure due to their comparatively larger ionic radii. The literature indicates that Rb^+^ (41 mAh/g) and Cs^+^ (28 mAh/g) have the lowest charge rates among the elements studied. Furthermore, the channel radius within the PBA crystal lattice is known to be approximately 1.6 Å, serving as a parameter to determine which elements can or cannot undergo ionic intercalation [80,81].

## 9. Different Uses of Prussian Blue Analogues

As Prussian Blues can exhibit different metal ion oxidation states and their frameworks can consist of charge-balancing vacancies [82], PBs have become attractive and versatile heterogeneous catalysts for various applications, such as wet chemical oxidation [83,84,85], electrochemical oxidation [86,87], electroreduction [46], reduction of nitrophenols [88], decomposition, and reduction of hydrogen peroxide [89,90], nitration of organic compounds [91], among others. Below are some of the uses for Prussian Blue.

### 9.1. Chemical Detection

Zearalenone (ZEN), is a major food contaminant mycotoxin that threatens human and animal health. An alkaline phosphatase (ALP)-activated dual-signal immunoassay was developed to detect ZEN in cornmeal. The test consists of catalyzing the free ZEN in the sample with the monoclonal antibody (McAb), which, through a series of reactions, catalyzes ascorbic acid 2-phosphate to produce L-ascorbic acid (AA), which is capable of converting the potassium ferricyanide (K_3_[Fe(CN)_6_]) to potassium ferricyanide (K_4_[Fe(CN)_6_]). This method causes the ferric(III) ion to react to promote the formation of Prussian Blue nanoparticles (PB NPs). Consequently, the solution produces a multicolor change and detection of the ZEN. Meanwhile, PB NPs have an absorption peak maximum of 700 nm and can be monitored by a UV–vis spectrometer. As an electron transfer medium, K_3_[Fe(CN)_6_] is gradually consumed along with the formation of PB NPs. Therefore, electrochemical detection is used to detect ZEN [92].

### 9.2. Treatment for Thallium Poisoning

Prussian Blue is the active pharmaceutical ingredient (API) in the drug Radiogardase^®^. This is the first approved medical countermeasure for the treatment of internal radioactive contamination that can result from radiological incidents, such as a nuclear attack or a dirty bomb.

PB, when administered orally, can increase the excretion of radioactive isotopes of cesium and thallium ions, and is also administered clinically in cases of suspected thallium poisoning [93]. The FDA (United States Food and Drug Administration) has approved the insoluble form (ferric hexacyanoferrate) of PB for the treatment of internal contamination by radio-thallium and radio-cesium and for thallium poisoning, with favorable results [94].

### 9.3. High Electrocatalytic Water Oxidation Activity

Prussian Blue Co/Fe coordination networks have recently been investigated for the oxidation catalysis of heterogeneous water. Despite its robustness and stability in both acid and neutral media, the current density is relatively low, its main drawback being the consequence of its low surface concentration. A novel synthetic approach using a pentacyanometalate-based metallopolymer for the preparation of amorphous Co/Fe coordination polymers is employed to overcome this problem. The surface concentration is improved approximately seven-fold, which also increases catalytic activity [87]. The current density of 1 mA/cm^2^ was obtained only at η = 510 mV, while the same current density can be obtained at higher overpotentials (>600 mV) with conventional Prussian Blue analogues.

### 9.4. Peroxide Detection

Prussian Blue (PB) particles with a size of approx. 5 nm were synthesized and immobilized in a multilayer structure as a strategy for the potential development of an amperometric transducer for oxidase enzyme-based biosensors. Multilayer films composed of PB and poly(allylamine hydrochloride) (PAH) were prepared layer by layer (LbL) and sequentially deposited. The process was carefully monitored using UV spectroscopy and cyclic voltammetry. Increased redox current peaks during layer-by-layer deposition demonstrated that charge propagation within the film occurs. The linear increase in UV–vis absorbance with the number of bilayers deposited indicates that well-organized systems have been made. ITO electrodes coated with PB/PAH films have been used successfully to detect H_2_O_2_, with sensitivity depending on the number of PB/PAH layers [44].

## 10. Electrochemical Metal Recovery Using Prussian Blue Analogue Materials

In the framework of metal recovery at an industrial scale, it is essential to cover basic considerations: (i) The identification of potential brine sources, and hopefully, the quantification of brine volume and time evolution of the ionic brine distribution; (ii) A rational selection of the ion to be recovered using adequate electrochemical cells with selected intercalation electrodes; (iii) Consider an economically viable source of energy near an industrial plant, principally in cases where brine sources are remote. Given the large availability of solar energy in arid regions and the successful implementation of large photovoltaic plants, this solar energy is in principle the main possible source of energy for potential metal recovery plants in Chile; however, solar *H*_2_ from seawater could also be an alternative energy supply; (v) Chile has extensive experience in recovery of various non-metallic salts using evaporation processes. In principle, one successful metal ion recovery process could comprise a combination of evaporation and electrochemical processes. Furthermore, due to the scarcity of water, it would be ideal that evaporative processes were designed so that the evaporated water be recovered by a solar still design; (vi) As a result of the intense mining activity in many parts of the world, there are many contaminated tailing ponds containing a variety of heavy metals that could also be treated using electrochemical processes. The current research and industrial experience have revealed the technical feasibility to recover many cations from brine solutions.

### 10.1. Sodium

Sodium has industrial use as a variety of different compounds and end-uses. Several researchers have proposed producing Na from a desalination concentrate solution [95]. The process of capturing and releasing the ions recovered from seawater is carried out via a four-step cycle proposed by La Mantia [7]. In this case, a cathodic electrode made with nanorods of Na_2−x_Mn_5_O_10_ (NMO) was used for the selective capture of Na^+^ ions due to its greater specific charge storage capacity (35 mA·h/g). It also has a low cost and low environmental impact. NMO was prepared using a polymer synthesis method, from which nanorods were obtained with a mean diameter of 300 nm and a length of approximately 2 µm. For the capture of Cl^−^ ions, the use of Ag is maintained, because it is stable with low insolubility for the capture of Cl^−^ ions. Several researchers have investigated the development of this technology For example, Yu Wang et al. [96,97] in 2014 and Slawomir Porada et al. [97,98] in 2016, who proposed the use of a pair of electrodes composed of NiHCF/MnO_2_. NiHCF is typically used in Na^+^ ion batteries, displaying excellent specific capacity, high power density, high energy density, and excellent stability in battery cycling. In 2017, Divyaraj Desai et al. [99] proposed KNaHCF as a cathodic electrode and Zn as an anodic electrode, for the selective capture of Na^+^ ions and Cl^−^ ions, obtaining specific capacities of 81 mAh/g in the cathode electrode and 828 mAh/g in the anode electrode. Jaehan Lee et al. [100] used NaNiHCF and NaFeHCF as cathode and anodic electrodes, respectively. These materials have the lowest production cost and excellent behavior against the intercalation of Na^+^ ions. In 2019, Do Hwan et al. [101,102] used a pair of CuHFC and Bi electrodes to expand the number of alternative low-cost materials that can be used for the recovery of Na^+^ ions and Cl^−^ ions from aqueous solutions. Table 2 shows the principal PBA materials used for Na^+^ capturing.

### 10.2. Potassium

The most popular potassium chemical products are potassium hydroxide (KOH) and potassium chloride (KCl)., These are widely used in various industrial fields, especially in chemical fertilizers or electronics. This element has a high commercial value; therefore, high purity (>99%) is required. Most potash operations employ conventional deep-well high-altitude brines to produce KCl, and, currently, KOH is produced commercially through a KCl electrolysis process. The KCl production process is carried out in potash plants, which use the precipitated salts of sylvinite (NaCl+KCl) and carnalite of potassium $\left(KCl\cdot Mg{Cl}_{2}\cdot 6H_{2}O\right)$ from a solar evaporation process carried out in the process of obtaining solutions rich in LiCl from highland brines, which contain large amounts of Na^+^ ions, these being KCl impurities, which are eliminated through crystallization processes. The K^+^ ion resources in seawater are extremely abundant. In 2019, Wei Shi et al. [103] presented the recovery of K^+^ ions from seawater through a deionization battery, which operates under the principle of the entropic mixing battery, using, in this case, FeHCF as a cathode material that operates selectively in the intercalation of the K^+^ ion. This material is stable during the ion absorption process, with a K^+^ ion retention efficiency of 86% in at least 150 cycles. Table 3 shows the principal PBA materials for K^+^ capturing.

### 10.3. Rubidium

Due to the relatively high price of Rb, several researchers have indicated that the extraction of Rb from seawater is economically viable [105]. In seawater reverse osmosis (SWRO) brine, Rb is present at a low concentration between 0.19 and 0.21 ppm with other predominant cations such as Na^+^, K^+^, Ca^2+^, Mg^2+^, and Li^+^ in highly saline conditions [106]. The principal battery materials used to recover Rb^+^ from saline electrolytes are members of the Prussia Blue Analogue family of materials. In 2016, Gayathri Naidu et al. proposed two different materials, namely, KCoHCF and KCuHCF(PAN), for the recovery of Rb^+^ from natural sources, considering its low concentration and the low selectivity of the agents [106,107,108]. At the same time, Nima Moazezi et al. studied the nanocomposite adsorbent using polyaniline (PANI) modified with CoHCF and prepared with the chemical precipitation method [109]. In 2017, Gayathri Naidu et al. proposed a new brine management method that recovered valuable metals using an integrated system of membrane distillation (MD) with KCuHCF(PAN) for the recovery of Rb^+^ and simultaneous SWRO brine volume reduction [100]. In 2018, T. Nur proposed the extraction of Rb^+^ from seawater using KCoHCF and ammonium molybdophosphate (AMP) adsorbents in the membrane adsorption hybrid system (MAHS) [110]. In 2023, Dai Quyet Truong et al. modified the structure of KCoFC by grafting zeolitic imidazole frameworks (ZIF) with KCoHCF to synthesize KCoHCF@ZIF [111]. Table 4 shows the principal PBA materials for capturing Rb^+^.

### 10.4. Cobalt

Cobalt is commonly used as a reactant in rechargeable batteries as $LiCoO_{2}$ or $LiNiMnCoO_{2}$ [114]. A quarter of Co metal produced around the world, especially that produced in Africa, is used in the construction of Li-ion batteries. As a result of this, the cost of Co has increased continuously in the last few years. The typical concentration of Co seawater is 0.1 mg/ton [53]. This transition metal is brittle, hard, a silver-gray color, and very toxic. The recovery of Co from recycled battery materials is mostly performed through pyrometallurgical and hydrometallurgical techniques, with the latter being most eco-friendly solution. The electrochemical method has many advantages in contrast to common techniques, with the following standing out: (i) high performance, (ii) easy operation, (iii) low cost, (iv) easy separation, and (v) being environmentally friendly. The selective recovery of Co^2+^ from aqueous solutions is possible by applying a reduction potential using battery materials such as CuHCF as a cathode. The electrochemical recovery of Co^2+^ from aqueous solutions was proposed for the first time in 2020 by Xinxin Long et al. [32].

### 10.5. Cesium

Commodity information on cesium, including pricing and usage data, is mostly unavailable. Despite the small market for cesium, several researchers have suggested that Cs could be extracted from SWRO brine for economic gain. The majority of studies on Cs extraction have evaluated ion exchange materials or liquid–liquid extraction processes and have not typically focused on obtaining a pure cesium product. This cation is a nucleotide with a radioactive half-life of 30.17 years. The selective removal of Cs can dramatically reduce the radioactivity of aqueous media from nuclear processing and is crucial for sustainable nuclear power. Battery materials such as NiHCF are very selective for monovalent ions in the order Li^+^ < Na^+^ < K^+^ < Rb^+^ < Cs^+^, and have selectivity towards Cs^+^ over a wide range of Cs^+^:Na^+^, even in the presence of 2000-fold excess Na^+^ [97]. At the same time, KCuFC, KZnFC, KFeFC, and KNiFC have also shown satisfactory results for Cs^+^ extraction from nuclear waste, with KCoFC being a specific and commercial material for Cs^+^ removal due to its high selectivity for radioactive materials like Cs [106]. Table 5 shows the principal PBA materials for capturing Cs^+^.

### 10.6. Other Metals

The typical concentration of rare metals in seawater is Y (0.3 mg/ton), Ti (1 mg/ton), Mn (2 mg/ton), V (2 mg/ton), U (3 mg/ton), Mo (10 mg/ton), B (4600 mg/ton), and Sr (8000 mg/ton). This implies that their concentration in geothermal water, altiplanic brine, and reverse osmosis brine could be higher than in seawater [53]. NiHCF has been used for the reversible insertion of alkaline earth divalent ions such as Mg^2+^, Ca^2+^, Sr^2+^, and Ba^2+^ with high rate capacities and high coulombic efficiencies, because of the electrode particle size, ionic conductivity, presence of zeolitic water, and open crystal structure of the active material. CuHCF was also used for the insertion of multivalent ions, such as Cu^2+^, Co^2+^, Pb^2+^, Nd^3+^, La^3+^, Sm^3+^, Y^3+^, and Ce^3+^, with very good reversibility of intercalation of many of these trivalent metals without much decay in their capacity after several cycles. For example, Y^3+^ sustained a good cyclability of more than 2000 cycles [70,73]. On the other hand, K-MHCF, where M is Cu, Mn, or Zn, was used for the recovery of Ca^2+^ from brackish water; whereas CuHCF and MnHCF showed promising performance for Ca^2+^ intercalation, ZnHCF was unstable and underwent rapid dissolution in contact with the CaCl_2_ solution [119].

## 11. Conclusions and Outlook

The exponential demand for strategic metals, essential for manufacturing various energy-generating, -storing, and -transporting devices, has been driven by active industrial interest and the global population’s increasing focus on green and renewable technologies, aiming to meet the world’s energy consumption requirements. The current understanding highlights the profound environmental impact and the limited availability of metals in the Earth’s crust, posing a potential threat to the extensive development of electronic technology, conversion devices, energy storage, and other industries due to the scarcity of raw materials. Conventional extraction processes, which are employed for metals such as Li, Cu, Ti, Pt, Ag, and Co, consume significant amounts of energy. Conversely, the recovery of metals like Mg, Na, K, and B relies on solar evaporation of high-altitude brines, resulting in substantial water loss without any existing means of recovery. Additionally, these processes are time-consuming, often exceeding 18 months, and are highly contingent on climatic conditions in the Chilean high-altitude region.

At the industrial level, various technologies exist for the recovery of ions from different sources, including natural or synthesized sources, mining effluents, and residual resources through environmental remediation. The most frequently employed methods include the following: (i) Electrodeposition: This technique involves an electrochemical system where an electric current is passed between two electrodes to facilitate the deposition of the solid metal on the cathode. It is widely regarded as an environmentally and economically efficient method, as it does not require additional reagents. The efficiency of electrodeposition typically ranges between 95% and 97% for metal recovery. (ii) Electrocoagulation: This method enables the simultaneous extraction of metal ions while generating hydroxyl and hydrogen groups through electron impacts on solid chains and dispersed oils. The effect leads to the solidification of all species into flocs, which can subsequently be processed and extracted from the solution. (iii) Electrodialysis: This technique involves the application of an electric current to separate ions by utilizing an ion exchange membrane. The ion exchange membrane plays a crucial role in generating ion transport, leading to efficient separation. Electrodialysis has demonstrated a high recovery efficiency, typically approaching 95%. (iv) Electro-electrodialysis: This method combines electrolysis and electrodialysis to enable the isolation of ions and their subsequent oxidation through the electrolysis process. By utilizing ion exchange membranes, it generates a continuous electric field that facilitates reactions at the electrodes. Electro-electrodialysis demonstrates a high recovery efficiency, typically approaching 98%. (v) Electrostatic separation: This method involves the application of a constant potential between two electrodes to selectively separate ions within an electrical domain. Through this process, ions can be effectively separated based on their charge. Electrostatic separation demonstrates a high recovery efficiency, typically approaching 95%. (vi) Cell Electroflotation: This is a process that involves the removal of ions, suspended metals particles or pollutants from a liquid medium using electrolysis. It utilizes the principles of electrochemistry and flotation to separate and concentrate suspended particles, such as contaminants, solids, or oils, from water or wastewater. Nevertheless, a comprehensive comparison of these methods under consistent operational parameters and energy consumption has not been conducted, making it challenging to determine whether the “electrochemical ion pumping” method surpasses metal extraction techniques from natural saline sources [120,121,122,123,124,125,126,127,128].

When comparing the saline solutions found in the Atacama Desert with other saline environments like the Dead Sea or the Great Salt Lake, several differences in NaCl concentration and ionic composition become apparent. Despite these variations, the salinity levels in the Atacama Desert are generally lower compared to the other two locations. The Dead Sea stands out as one of the saltiest bodies of water on Earth, boasting salinity levels approximately 10 times higher than that of seawater elsewhere. Similarly, the Great Salt Lake in Utah, USA, also exhibits higher salinity levels compared to the Atacama Desert. In terms of chemical composition, the brine in the Atacama Desert primarily consists of NaCl, KCl, MgCl_2_, and other mineral salts. On the other hand, the dominant salts in the Dead Sea are MgCl_2_, NaCl, and KCl. The Great Salt Lake contains a diverse array of salts, including NaCl, MgCl_2_, CaCl_2_, and KCl.

In today’s context, the generation of alternative and environmentally friendly technological innovations holds paramount importance, aiming to minimize ecological impact while effectively addressing our needs. In this regard, a promising solution arises in the form of electrochemical technology for metal recovery, capable of capturing or retrieving specific ions from saturated solutions and subsequently concentrating them in another solution. This innovative approach finds its foundation in the entropic mixing battery, which operates on a relatively new electrochemical technique known as “electrochemical ion pumping”. This method offers great potential in achieving sustainable metal recovery while mitigating environmental repercussions. This versatile technology has the potential not only to recover desired ions from various natural saline sources like seawater, geothermal waters, high-altitude brines, and brines from osmosis plants, but also to extract metals from the solution regardless of the solutions pH. Based on these capabilities, futuristic applications can be envisioned in multiple domains, including the recycling of batteries containing Li, Na, Mg, Zn, and Al, as well as in the recycling of solar panels and electrolyzers. Moreover, it holds promise in mining processes, specifically in the direct recovery of Cu^2+^/Cu^+^ from the acidic and multicomponent solution generated during the leaching process, commonly referred to as “Pregnant Leach Solution” (PLS). PLS denotes metal-laden acidic water produced from heap leaching. The technology’s adaptability positions it as a valuable tool in various industries, facilitating efficient metal recovery and contributing to sustainable resource utilization.

The “electrochemical ion pumping” method offers several distinct advantages over conventional processes, primarily stemming from the following factors: (i) The estimated potential environmental impact is significantly lower compared to conventional marine mining practices; (ii) The method can be precisely targeted towards specific ions through the implementation of highly selective electrodes, allowing for tailored extraction; (iii) Energy consumption and evaporative water losses can be minimized by employing complementary conventional energy and water recovery technologies in conjunction with the electrochemical method; and (iv) The technology for ion pump devices is well-established, enabling ease of implementation. Additionally, it can be readily extended to address the treatment of highly toxic mining wastes that are currently stored in large-scale ponds. These advantages highlight the potential of the “electrochemical ion pumping” method to revolutionize mining processes, ensuring reduced environmental impact, enhanced selectivity, improved resource efficiency, and the remediation of hazardous mining waste materials.

Prussian Blues have gained significant attention as cathode materials for the recovery of various metals in solution, due to their exceptional electrochemical performance and simple and efficient synthetic methods. This attribute becomes particularly crucial when considering industrial-scale implementation, as these materials can be classified as cost-effective cathode options. The simplest synthesis method for Prussian Blues involves co-precipitation, which relies on straightforward unitary mixing operations rather than complex mixing and calcination processes found in the synthesis of Li spinels like LiMn_2_O_4_ or LiFePO_4_. This inherent simplicity in Prussian Blue synthesis contributes to their attractiveness as low-cost cathode materials, bolstering their potential for large-scale industrial applications. In the realm of electrode materials, Prussian Blue Analogues (PBAs) hold significant promise, displaying commendable redox properties and distinctive standard potentials. They can selectively intercalate ions, considering the available intermolecular space and the electro-affinity of the targeted ion. Determining the optimal operational parameters for Prussian Blue Analogue (PBA) materials can be challenging due to the multitude of available synthesis methods. To address this, it is imperative to establish a standardized methodology that encompasses the following essential parameters: (i) selectivity: the ability to preferentially intercalate specific ions of interest; (ii) absorption capacity: the maximum amount of ions that can be absorbed by the PBA material; (iii) energy consumption: the energy required for the intercalation and recovery processes; (iv) concentration increase per cycle: the extent to which the concentration of the target ions can be increased in each intercalation cycle; (v) overall and per-cycle efficiency: the effectiveness of the process in terms of ion recovery, taking into account losses and energy consumption; and (vi) product purity: the degree of purity achieved in the final recovered metal product.

Moreover, the efficiency of metal recovery from aqueous solutions is closely tied to several factors, including (i) the choice of cathode material, (ii) the ionic radii of the target metal ions, (iii) the electrode’s porosity, influencing ion diffusion and intercalation, (iv) the diameter of the intercalation channels within the electrode, and (v) the specific synthesis method employed to create the active material. By comprehensively understanding and optimizing these parameters, the development of efficient and effective metal recovery processes utilizing PBAs can be realized. The synthesis approach employed for the cathode material plays a crucial role in determining the ion adsorption capacity within its crystalline structure. Consequently, higher electrochemical performance is typically associated with increased adsorption capacity, which varies depending on the specific PBA. PBAs, in general, exhibit high porosity, conductivity, and optimized electrochemical properties.

Numerous studies have played a crucial role in investigating the ladder mechanism in PBAs, aiming to estimate energy barriers by simulating energies in assumed positions for intercalation sites and wall sites. Similar approaches are employed for studying other crystal types as well. The complexity of intercalation landscapes, encompassing both intercalation positions and transition paths, significantly impacts material performance. The key lies in identifying intercalation sites and the main transition pathways within the intricate geometry of PBAs to calculate the volume available for ion movement. The discovered energy landscape emphasizes the importance of examining local interactions to determine the favorability of transitions. Understanding favorable sites and the fastest routes between them is vital for comprehending diffusion in complex materials. Defects, which can vary across materials, also offer intriguing perspectives. Interestingly, diffusion occurs more rapidly at the corners of vacant unit cells compared to unit cells without vacancies. This is due to the increased stability provided by cavities enclosing ions from all sides at intercalation sites. By employing the self-consistent, mean-field method for simulating ion hopping, we can generate a unique prediction for the macroscopic diffusion constant based on energy levels obtained from quantum-level simulations. Additionally, each level of simulation introduces new challenges. Combining quantum-level output with a macroscopic prediction of tortuosity enables estimation of the effects of defect density. Future work could expand this methodology to different scales, such as using quantum-level simulations to estimate intercalation energies and conducting calculations to simulate performance at the device level.

However, there exists significant disagreement in the methods employed to concentrate the recovered product over time. This is due to the decrease in adsorption capacity as the intercalation and deintercalation process progresses with each cycle. Alongside the technological considerations regarding electrode efficiency, several factors must be considered when aiming for the industrial-scale transition of metal recovery processes. Examples of such factors include electrode reactivation methods, characteristics of the unit cell, a comprehensive cell design matrix, selection of corrosion-resistant materials, optimal location for brine source sites, plant design for the specific industry, proximity to an energy source, and other relevant considerations. Additionally, a thorough understanding of operating costs (OPEX) and their correlation with the number of operating cycles is crucial for effective decision-making in scaling up metal recovery operations.

## Figures and Tables

**Table 1:** Methods of obtaining Prussian Blue with their respective synthesis [36,37,39,40].

Methods	Preparation Processes	Advantages	Disadvantages
Coprecipitation (PBA)	Mix metal ions with K_3_[M(CN)_6_] (M = Ni, Co, Fe, etc.)	Simple and low cost	Rapid nucleation leading to PBA with irregular morphology
Etching (PBA)	Use K_3_[M(CN)_6_] (M = Ni, Co, Fe, etc.) to etch hydroxides/metal oxides	Effectively control nucleation rate, resulting in well-structured PBAs	Need to prepare hydroxides/metal oxides or fix them first
Solution phase reaction (derivatives)	React PBA precursors/templates with S or Se sources (Na_2_S, (NH_4_)_2_ MoS_4_, Se powder, etc.) under hydrothermal/solvothermal conditions	Low energy consumption (the reaction temperature is always below 200 °C)	Derivatized catalysts have poor catalytic performance
Gas–solid reaction (derivatives)	Heat treat PBA precursors/templates with sulfur powder, Se powder, or NaH_2_PO_2_ in the tube furnace under an inert gas atmosphere	Obtaining high conductivity graphitized carbon; derived catalysts show excellent catalytic performance	High energy consumption (reaction temperature usually exceeds 600 °C)

**Table 2:** PBA materials for Na^+^ recovery from aqueous solutions.

PBA	Electrolyte Type	Capacity (mAh/g)	% Recovery of Removal	Ref.
NiHCF ^a^	Brackish Water	59	89	[98]
CuHCF	-	-	-	[101,102]
KNaHCF ^b^	Seawater	35	85	[99]
KNaHCF ^b^	Hypersaline Brine	-	86	[99]
NaNiHCF	Seawater	-	40	[100]
NaFeHCF	Seawater	-	40	[100]

^a^ BET area of NiHCF: 15 m^2^/g, minimum energy consumption: 47 kJ/mol-salt. ^b^ energy consumption (Seawater): 4.08 Wh/mol; energy consumption (Hypersaline brine): 4.08 Wh/mol; desalination flux (4.7 mol/m^2^h).

**Table 3:** PBA materials for K^+^ recovery from aqueous solutions.

PBA	Electrolyte Type	Capacity (mAh/g)	% Recovery of Removal	Ref.
KFeHCF ^a^	KCl solution	-	18.7	[104]
FeHCF ^b^	Seawater	-	69.6	[103]

^a^ Current density: 0.3 mA/cm^2^. ^b^ Recovery capacity: 177 µmoL/g (under the discharge stage from 0.8 to 0 V) and intercalation of K^+^ achieves a high specific capacity of 220.0 F/g.

**Table 4:** PBA materials for Rb^+^ recovery from aqueous solutions.

BA	Electrolyte Type	Capacity (mAh/g)	% Recovery of Removal	Ref.
KCuHCF(PAN)	SWRO Brine	-	95	[100,108,110]
KCoHCF	SWRO Brine	-	74	[110]
KCoHCF	Boron Industrial Waste	-	66	[112]
CoHCF(PANI) ^a^	Aqueous Solutions	-	98	[109]
KNiHCF	Salt Lake Brine	-	99.97	[113]
KCoFC@ZIF ^b^	Seawater	-	45	[111]

^a^ The sorption of Rb^+^ onto the CoHCF(PANI) is endothermic and spontaneous in 240 h operation. ^b^ The grafted ZIF layer works as a catalyst and increases the surface area of the material for Rb^+^ recovery, but it reduces the presence of K^+^ ions in seawater product by about 45%.

**Table 5:** PBA materials for Cs^+^ recovery from aqueous solutions.

PBA	Electrolyte Type	Capacity (mAh/g)	% Recovery of Removal	Ref.
NiHCF ^a^	Radioactive wastewater	-	95.3	[115,116,117]
ZnHCF	Salt lake brine and geothermal water	-	98.6	[118]

^a^ The loss of capacity for the CNT–PANI–NiHCF films is the smallest after about 500 cycles, retaining 92% of the initial capacity.

## Data Availability

The supporting data are available from the corresponding authors.

## References

[1] Gibert O., Valderrama C., Peterkóva M., Cortina J.L. (2010). Evaluation of Selective Sorbents for the Extraction of Valuable Metal Ions (Cs, Rb, Li, U) from Reverse Osmosis Rejected Brine. Solvent Extr. Ion Exch..

[2] Xu P., Hong J., Qian X., Xu Z., Xia H., Tao X., Xu Z., Ni Q.-Q. (2021). Materials for Lithium Recovery from Salt Lake Brine. J. Mater. Sci..

[3] Kanoh H., Ooi K., Miyai Y., Katoh S. (1993). Electrochemical Recovery of Lithium Ions in the Aqueous Phase. Sep. Sci. Technol..

[4] Pasta M., Battistel A., Mantia F. (2012). La Batteries for Lithium Recovery from Brines. Energy Environ. Sci..

[5] Lee J., Yu S.-H., Kim C., Sung Y.-E., Yoon J. (2013). Highly Selective Lithium Recovery from Brine Using a λ-MnO_2_–Ag Battery. Phys. Chem. Chem. Phys..

[6] Ooi K., Miyai Y., Katoh S., Maeda H., Abe M. (1989). Topotactic lithium(1+) insertion to .lambda.-manganese dioxide in the aqueous phase. Langmuir.

[7] La Mantia F., Pasta M., Deshazer H.D., Logan B.E., Cui Y. (2011). Batteries for Efficient Energy Extraction from a Water Salinity Difference. Nanoletters.

[8] Pasta M., Wessells C.D., Cui Y., La Mantia F. (2012). A Desalination Battery. Nanoletters.

[9] Md Hasan K.N., Khai T., Kannan R., Zakaria Z. (2017). Harnessing ‘Blue Energy’: A Review on Techniques and Preliminary Analysis. MATEC Web Conf..

[10] Lee J., Yoon H., Lee J., Kim T., Yoon J. (2017). Extraction of Salinity-Gradient Energy by a Hybrid Capacitive-Mixing System. ChemSusChem.

[11] Marino M., Misuri L., Carati A., Brogioli D. (2014). Proof-of-Concept of a Zinc-Silver Battery for the Extraction of Energy from a Concentration Difference. Energies.

[12] Brogioli D., Ziano R., Rica R., Salerno D., Kozynchenko O., Hamelers H.V.M., Mantegazza F. (2012). Exploiting the Spontaneous Potential of the Electrodes Used in the Capacitive Mixing Technique for the Extraction of Energy from Salinity Difference. Energy Environ. Sci..

[13] Kim T., Rahimi M., Logan B., Gorski C. (2016). Harvesting Energy from Salinity Differences Using Battery Electrodes in a Concentration Flow Cell. Environ. Sci. Technol..

[14] Morais W., Gomes W., Huguenin F. (2016). Neutralization Pseudocapacitors: An Acid-Base Machine. J. Phys. Chem. C.

[15] Brogioli D. (2009). Extracting Renewable Energy from a Salinity Difference Using a Capacitor. Phys. Rev. Lett..

[16] Marino M., Misuri L., Ruffo R., Brogioli D. (2015). Electrode Kinetics in the “Capacitive Mixing” and “Battery Mixing” Techniques for Energy Production from Salinity Differences. Electrochim. Acta.

[17] Kim T., Rahimi M., Logan B., Gorski C. (2016). Evaluating Battery-like Reactions to Harvest Energy from Salinity Differences Using Ammonium Bicarbonate Salt Solutions. ChemSusChem.

[18] Sharma K., Kim Y.H., Yiacoumi S., Gabitto J., Bilheux H.Z., Santodonato L.J., Mayes R.T., Dai S., Tsouris C. (2016). Analysis and Simulation of a Blue Energy Cycle. Renew. Energy.

[19] Tan G., Zhu X. (2020). Polyelectrolyte-Coated CuHCF and BiOCl Electrodes for Efficient Salinity Gradient Energy Recovery in Capacitive Mixing. Energy Technol..

[20] Logan B., Elimelech M. (2012). Membrane-Based Processes for Sustainable Power Generation Using Water. Nature.

[21] Sales B.B., Liu F., Schaetzle O., Buisman C.J.N., Hamelers H.V.M. (2012). Electrochemical Characterization of a Supercapacitor Flow Cell for Power Production from Salinity Gradients. Electrochim. Acta.

[22] Jia Z., Wang B., Song S., Fan Y. (2013). A Membrane-Less Na Ion Battery-Based CAPMIX Cell for Energy Extraction Using Water Salinity Gradients. RSC Adv..

[23] Post J.W., Hamelers H.V.M., Buisman C.J.N. (2008). Energy Recovery from Controlled Mixing Salt and Fresh Water with a Reverse Electrodialysis System. Environ. Sci. Technol..

[24] Jia Z., Wang B., Song S., Fan Y. (2014). Blue Energy: Current Technologies for Sustainable Power Generation from Water Salinity Gradient. Renew. Sustain. Energy Rev..

[25] Haj Mohammad Hosein Tehrani S., Seyedsadjadi S.A., Ghaffarinejad A. (2015). Application of Electrodeposited Cobalt Hexacyanoferrate Film to Extract Energy from Water Salinity Gradients. RSC Adv..

[26] Trócoli R., Erinmwingbovo C., LaMantia F. (2016). Optimized Lithium Recovery from Brines by Using an Electrochemical Ion-Pumping Process Based on λ-MnO2 and Nickel Hexacyanoferrate. ChemElectroChem.

[27] Zhou G., Chen L., Chao Y., Li X., Luo G., Zhu W. (2021). Progress in Electrochemical Lithium Ion Pumping for Lithium Recovery. J. Energy Chem..

[28] Battistel A., Palagonia M.S., Brogioli D., La Mantia F., Trócoli R. (2020). Electrochemical Methods for Lithium Recovery: A Comprehensive and Critical Review. Adv. Mater..

[29] Lawagon C., Nisola G., Cuevas R., Kim H., Lee S.-P., Chung W.-J. (2018). Li 1- x Ni 0.33 Co 1/3 Mn 1/3 O 2/Ag for Electrochemical Lithium Recovery from Brine. Chem. Eng. J..

[30] Jeong J.M., Rhee K.Y., Park S.J. (2015). Effect of Chemical Treatments on Lithium Recovery Process of Activated Carbons. J. Ind. Eng. Chem..

[31] Zhao M.Y., Ji Z.Y., Zhang Y.G., Guo Z.Y., Zhao Y.Y., Liu J., Yuan J.S. (2017). Study on Lithium Extraction from Brines Based on LiMn2O4/Li1-XMn2O4 by Electrochemical Method. Electrochim. Acta.

[32] Long X., Chen R., Tan J., Lu Y., Wang J., Huang T., Lei Q. (2020). Electrochemical Recovery of Cobalt Using Nanoparticles Film of Copper Hexacyanoferrates from Aqueous Solution. J. Hazard. Mater..

[33] Wang L., Meng C.G., Ma W. (2009). Study on Li+ Uptake by Lithium Ion-Sieve via the PH Technique. Colloids Surfaces A Physicochem. Eng. Asp..

[34] Hu M., Ishihara S., Ariga K., Imura M., Yamauchi Y. (2013). Kinetically Controlled Crystallization for Synthesis of Monodispersed Coordination Polymer Nanocubes and Their Self-Assembly to Periodic Arrangements. Chem. Eur. J..

[35] Hu M., Torad N.L., Yamauchi Y. (2012). Preparation of Various Prussian Blue Analogue Hollow Nanocubes with Single Crystalline Shells. Eur. J. Inorg. Chem..

[36] Doumic L.I., Salierno G.L., Cassanello M.C., Haure P.M., Ayude M.A. (2013). Prussian Blue onto Activated Carbon as a Catalyst for Heterogeneous Fenton-like Processes. IJCEA.

[37] Vipin A.K., Fugetsu B., Sakata I., Isogai A., Endo M., Li M., Dresselhaus M.S. (2016). Cellulose Nanofiber Backboned Prussian Blue Nanoparticles as Powerful Adsorbents for the Selective Elimination of Radioactive Cesium. Sci. Rep..

[38] Yang Y., Lun Z., Xia G., Zheng F., He M., Chen Q. (2015). Non-Precious Alloy Encapsulated in Nitrogen-Doped Graphene Layers Derived from MOFs as an Active and Durable Hydrogen Evolution Reaction Catalyst. Energy Environ. Sci..

[39] Wang Y., Ma J., Wang J., Chen S., Wang H., Zhang J. (2019). Interfacial Scaffolding Preparation of Hierarchical PBA-Based Derivative Electrocatalysts for Efficient Water Splitting. Adv. Energy Mater..

[40] Yu X., Yu L., Wu H., Lou X. (2015). Formation of Nickel Sulfide Nanoframes from Metal–Organic Frameworks with Enhanced Pseudocapacitive and Electrocatalytic Properties. Angew. Chemie Int. Ed..

[41] Zhang X., Li C., Si T., Lei H., Wei C., Sun Y., Zhan T., Liu Q., Guo J. (2018). FeNi Cubic Cage@N-Doped Carbon Coupled with N-Doped Graphene toward Efficient Electrochemical Water Oxidation. ACS Sustain. Chem. Eng..

[42] Guo H., Li T., Chen W., Liu L., Yang X., Wang Y., Guo Y. (2014). General Design of Hollow Porous CoFe2O4 Nanocubes from Metal–Organic Frameworks with Extraordinary Lithium Storage. Nanoscale.

[43] Shiba F. (2010). Preparation of Monodisperse Prussian Blue Nanoparticles via Reduction Process with Citric Acid. Colloids Surfaces A Physicochem. Eng. Asp..

[44] Fiorito P.A., Gonçales V.R., Ponzio E.A., de Torresi S.I.C. (2005). Synthesis, Characterization and Immobilization of Prussian Blue Nanoparticles. A Potential Tool for Biosensing Devices. Chem. Commun..

[45] Wu C.H., Chiu Y.T., Lin K.Y.A. (2018). Macrosphere-Supported Nanoscale Prussian Blue Analogues Prepared via Self-Assembly as Multi-Functional Heterogeneous Catalysts for Aqueous Oxidative and Reductive Reactions. Sep. Purif. Technol..

[46] Bai J., Qi B., Ndamanisha J.C., Guo L. (2009). ping Ordered Mesoporous Carbon-Supported Prussian Blue: Characterization and Electrocatalytic Properties. Microporous Mesoporous Mater..

[47] Senthilkumar S.T., Go W., Han J., Thuy L.P.T., Kishor K., Kim Y., Kim Y. (2019). Emergence of Rechargeable Seawater Batteries. J. Mater. Chem. A.

[48] Bardi U. (2010). Extracting Minerals from Seawater: An Energy Analysis. Sustainability.

[49] Cisternas L.A., Gálvez E.D. (2018). The Use of Seawater in Mining. Miner. Process. Extr. Metall. Rev..

[50] Diallo M.S., Rao Kotte M., Cho M. (2015). Mining Critical Metals and Elements from Seawater: Opportunities and Challenges. Enviromental Sci. Technol..

[51] Tapia J., González R., Townley B., Oliveros V., Álvarez F., Aguilar G., Menzies A., Calderón M. (2018). Geology and Geochemistry of the Atacama Desert. Antonie Leeuwenhoek.

[52] Jia Z., Wang B., Wang Y. (2014). Copper Hexacyanoferrate with a Well-Defined Open Framework as a Positive Electrode for Aqueous Zinc Ion Batteries. Mater. Chem. Phys..

[53] Nakazawa N., Tamada M., Seko N., Ooi K., Akagawa S. Experimental Studies on Rare Metal Collection from Seawater. Proceedings of the Ninth ISOPE Ocean Mining Symposium.

[54] Roberts D.A., Johnston E.L., Knott N.A. (2010). Impacts of Desalination Plant Discharges on the Marine Environment: A Critical Review of Published Studies. Water Res..

[55] Intaranont N., Garcia-Araez N., Hector A., Milton J., Owen J. (2014). Selective Lithium Extraction from Brines by Chemical Reaction with Battery Materials. J. Mater. Chem. A.

[56] Schmidt A., Mestmäcker F., Brückner L., Elwert T., Strube J. (2019). Liquid-Liquid Extraction and Chromatography Process Routes for the Purification of Lithium. Mater. Sci. Forum.

[57] Chitrakar R., Kanoh H., Miyai Y., Ooi K. (2001). Recovery of Lithium from Seawater Using Manganese OxideAdsorbent (H_1.6_Mn_1.6_O_4_) Derived from Li_1.6_Mn_1.6_O_4_. Ind. Eng. Chem. Res..

[58] Miyai Y., Ooi K., Katoh S. (1988). Recovery of Lithium from Seawater Using a New Type of Ion-Sieve Adsorbent Based on MgMn_2_O_4_. Sep. Sci. Technol..

[59] Gallup D.L. (1998). Geochemistry of Geothermal Fluids and Well Scales, and Potential for Mineral Recovery. Ore Geol. Rev..

[60] Hoshino T. (2013). Preliminary Studies of Lithium Recovery Technology from Seawater by Electrodialysis Using Ionic Liquid Membrane. Desalination.

[61] Swain B. (2017). Recovery and Recycling of Lithium: A Review. Sep. Purif. Technol..

[62] Evans R.K. (2008). An Abundance of Lithium. https://api.semanticscholar.org/CorpusID:10640157.

[63] Carmona V., Pueyo J.J., Taberner C., Chong G., Thirlwall M. (2000). Solute Inputs in the Salar de Atacama (N. Chile). J. Geochemical Explor..

[64] Leybourne M.I., Cameron E.M. (2006). Composition of Groundwaters Associated with Porphyry-Cu Deposits, Atacama Desert, Chile: Elemental and Isotopic Constraints on Water Sources and Water-Rock Reactions. Geochim. Cosmochim. Acta.

[65] Han L., Tang P., Reyes-Carmona A., Rodriguez-Garcia B., Torrens M., Morante J.R., Arbiol J., Galan-Mascaros J.R. (2016). Enhanced Activity and Acid PH Stability of Prussian Blue-Type Oxygen Evolution Electrocatalysts Processed by Chemical Etching. J. Am. Chem. Soc..

[66] Isfahani V.B., Memarian N., Rezagholipour H., Arab A., Silva M.M. (2019). Electrochimica Acta The Physical and Electrochromic Properties of Prussian Blue Thin Fi Lms Electrodeposited on ITO Electrodes. Electrochim. Acta.

[67] Guo J., Hao X., Ma X., Zhang Z., Liu S. (2008). Electrochemical Characterization of Ion Selectivity in Electrodeposited Nickel Hexacyanoferrate Thin Films. J. Univ. Sci. Technol. Beijing Miner. Metall. Mater..

[68] Ho K.-C., Lin C.-L. (2001). A Novel Potassium Ion Sensing Based on Prussian Blue Thin Films. Sensors Actuators B.

[69] Zakaria M.B., Belik A.A., Nagata T., Takei T., Tominaka S., Chikyow T. (2019). Molecular Magnetic Thin Films Made from Ni-Co Prussian Blue Analogue Anchored on Silicon Wafers. J. Magn. Magn. Mater..

[70] Guduru R.K., Icaza J.C. (2016). A Brief Review on Multivalent Intercalation Batteries with Aqueous Electrolytes. Nanomaterials.

[71] Yang N., Sun H. (2007). Biocoordination Chemistry of Bismuth: Recent Advances. Coord. Chem. Rev..

[72] Ma F., Li Q., Wang T., Zhang H., Wu G. (2017). Energy Storage Materials Derived from Prussian Blue Analogues. Sci. Bull..

[73] Wang R.Y., Shyam B., Stone K.H., Weker J.N., Pasta M., Lee H., Toney M.F., Cui Y. (2015). Reversible Multivalent (Monovalent, Divalent, Trivalent) Ion Insertion in Open Framework Materials. Adv. Energy Mater..

[74] Galleguillos F., Cáceres L., Maxwell L., Soliz Á. (2020). Electrochemical Ion Pumping Device for Blue Energy Recovery: Mixing Entropy Battery. Appl. Sci..

[75] Chen F.P., Jin G.P., Peng S.Y., Liu X.D., Tian J.J. (2016). Recovery of Cesium from Residual Salt Lake Brine in Qarham Playa of Qaidam Basin with Prussian Blue Functionalized Graphene/Carbon Fibers Composite. Colloids Surfaces A Physicochem. Eng. Asp..

[76] Trocoli R., Bidhendi G.K., Mantia F. (2016). La Lithium Recovery by Means of Electrochemical Ion Pumping: A Comparison between Salt Capturing and Selective Exchange. J. Phys. Condens. Matter.

[77] Gomes W.J.A.S., De Oliveira C., Huguenin F. (2015). Energy Harvesting by Nickel Prussian Blue Analogue Electrode in Neutralization and Mixing Entropy Batteries. Langmuir.

[78] Ye M., Pasta M., Xie X., Dubrawski K.L., Xu J., Liu C., Cui Y., Criddle C.S. (2019). Charge-Free Mixing Entropy Battery Enabled by Low-Cost Electrode Materials. ACS Omega.

[79] Tojo T., Sugiura Y., Inada R., Sakurai Y. (2016). Reversible Calcium Ion Batteries Using a Dehydrated Prussian Blue Analogue Cathode. Electrochim. Acta.

[80] Phadke S., Mysyk R., Anouti M. (2020). Effect of Cation (Li+, Na+, K+, Rb+, Cs+) in Aqueous Electrolyte on the Electrochemical Redox of Prussian Blue Analogue (PBA) Cathodes. J. Energy Chem..

[81] Shannon R.D. (1976). Revised Effective Ionic Radii and Systematic Studies of Interatomic Distances in Halides and Chalcogenides. Acta Crystallogr. Sect. A.

[82] Karadas F., El-Faki H., Deniz E., Yavuz C.T., Aparicio S., Atilhan M. (2012). CO_2_ Adsorption Studies on Prussian Blue Analogues. Microporous Mesoporous Mater..

[83] Li X., Liu J., Rykov A.I., Han H., Jin C., Liu X., Wang J. (2015). Excellent Photo-Fenton Catalysts of Fe-Co Prussian Blue Analogues and Their Reaction Mechanism Study. Appl. Catal. B Environ..

[84] Li X., Wang J., Rykov A.I., Sharma V.K., Wei H., Jin C., Liu X., Li M., Yu S., Sun C. (2015). Prussian Blue/TiO2 Nanocomposites as a Heterogeneous Photo-Fenton Catalyst for Degradation of Organic Pollutants in Water. Catal. Sci. Technol..

[85] Lin K.-Y.A., Chen B.-J., Chen C.-K. (2016). Evaluating Prussian Blue Analogues MII3[MIII(CN)6]2 (MII = Co, Cu, Fe, Mn, Ni; MIII = Co, Fe) as Activators for Peroxymonosulfate in Water. RSC Adv..

[86] Pintado S., Goberna-Ferrón S., Escudero-Adán E.C., Galán-Mascarós J.R. (2013). Fast and Persistent Electrocatalytic Water Oxidation by Co–Fe Prussian Blue Coordination Polymers. J. Am. Chem. Soc..

[87] Aksoy M., Nune S.V.K., Karadas F. (2016). A Novel Synthetic Route for the Preparation of an Amorphous Co/Fe Prussian Blue Coordination Compound with High Electrocatalytic Water Oxidation Activity. Inorg. Chem..

[88] Chen R., Zhang Q., Gu Y., Tang L., Li C., Zhang Z. (2015). One-Pot Green Synthesis of Prussian Blue Nanocubes Decorated Reduced Graphene Oxide Using Mushroom Extract for Efficient 4-Nitrophenol Reduction. Anal. Chim. Acta.

[89] Uyanik G., Pekin B. (1970). The Deactivation of Prussian Blue Heterogeneous Catalyst during the Decomposition of H_2_O_2_ in Aqueous Alkaline Solution. J. Catal..

[90] Jin R., Li L., Lian Y., Xu X., Zhao F. (2012). Layered Double Hydroxide Supported Prussian Blue Nanocomposites for Electrocatalytic Reduction of H_2_O_2_. Anal. Methods.

[91] Pasnoori S., Kamatala C.R., Mukka S.K., Kancharla R.R. (2014). Prussian Blue as an Eco-Friendly Catalyst for Selective Nitration of Organic Compounds Under Conventional and Nonconventional Conditions. Synth. React. Inorg. Met. Nano-Metal Chem..

[92] Shang C., Li Y., Zhang Q., Tang S., Tang X., Ren H., Hu P., Lu S., Li P., Zhou Y. (2022). Alkaline Phosphatase-Triggered Dual-Signal Immunoassay for Colorimetric and Electrochemical Detection of Zearalenone in Cornmeal. Sens. Actuators B Chem..

[93] Atsmon J., Taliansky E., Neufeld M.Y., Landau M. (2000). Thallium Poisoning in Israel. Am. J. Med. Sci..

[94] Faustino P.J., Brown A., Lowry B., Yang Y., Wang Y., Khan M.A., Dunbar K.R., Mohammad A. (2019). Quantitative Evaluation of the Thallium Binding of Soluble and Insoluble Prussian Blue Hexacyanoferrate Analogs: A Scientific Comparison Based on Their Critical Quality Attributes. Int. J. Pharm..

[95] Shahmansouri A., Min J., Jin L., Bellona C. (2015). Feasibility of Extracting Valuable Minerals from Desalination Concentrate: A Comprehensive Literature Review. J. Clean. Prod..

[96] Wang Y., Chen Q. (2014). Dual-Layer-Structured Nickel Hexacyanoferrate/MnO2 Composite as a High-Energy Supercapacitive Material Based on the Complementarity and Interlayer Concentration Enhancement Effect. ACS Appl. Mater. Interfaces.

[97] Su X., Hatton T.A. (2017). Redox-Electrodes for Selective Electrochemical Separations. Adv. Colloid Interface Sci..

[98] Porada S., Shrivastava A., Bukowska P., Biesheuvel P.M., Smith K.C. (2017). Nickel Hexacyanoferrate Electrodes for Continuous Cation Intercalation Desalination of Brackish Water. Electrochim. Acta.

[99] Desai D., Beh E.S., Sahu S., Vedharathinam V., Van Overmeere Q., De Lannoy C.F., Jose A.P., Völkel A.R., Rivest J.B. (2018). Electrochemical Desalination of Seawater and Hypersaline Brines with Coupled Electricity Storage. ACS Energy Lett..

[100] Naidu G., Jeong S., Johir M.A.H., Fane A.G., Kandasamy J., Vigneswaran S. (2017). Rubidium Extraction from Seawater Brine by an Integrated Membrane Distillation-Selective Sorption System. Water Res..

[101] Nam D.H., Choi K.S. (2017). Bismuth as a New Chloride-Storage Electrode Enabling the Construction of a Practical High Capacity Desalination Battery. J. Am. Chem. Soc..

[102] Nam D.H., Choi K.S. (2018). Electrochemical Desalination Using Bi/BiOCl Electrodialysis Cells. ACS Sustain. Chem. Eng..

[103] Shi W., Nie P., Shang X., Yang J., Xie Z., Xu R., Liu J. (2019). Berlin Green-Based Battery Deionization-Highly Selective Potassium Recovery in Seawater. Electrochim. Acta.

[104] Yoon H., Lee J., Kim S., Yoon J. (2018). Electrochemical Sodium Ion Impurity Removal System for Producing High Purity KCl. Hydrometallurgy.

[105] Pérez-González A., Urtiaga A.M., Ibáñez R., Ortiz I. (2012). State of the Art and Review on the Treatment Technologies of Water Reverse Osmosis Concentrates. Water Res..

[106] Nur T., Naidu G., Loganathan P., Kandasamy J., Vigneswaran S. (2016). Rubidium Recovery Using Potassium Cobalt Hexacyanoferrate Sorbent. Desalin. Water Treat..

[107] Naidu G., Loganathan P., Jeong S., Johir M.A.H., To V.H.P., Kandasamy J., Vigneswaran S. (2016). Rubidium Extraction Using an Organic Polymer Encapsulated Potassium Copper Hexacyanoferrate Sorbent. Chem. Eng. J..

[108] Naidu G., Nur T., Loganathan P., Kandasamy J., Vigneswaran S. (2016). Selective Sorption of Rubidium by Potassium Cobalt Hexacyanoferrate. Sep. Purif. Technol..

[109] Moazezi N., Moosavian M.A. (2016). Removal of Rubidium Ions by Polyaniline Nanocomposites Modified with Cobalt-Prussian Blue Analogues. J. Environ. Chem. Eng..

[110] Nur T., Loganathan P., Johir M.A.H., Kandasamy J., Vigneswaran S. (2018). Removing Rubidium Using Potassium Cobalt Hexacyanoferrate in the Membrane Adsorption Hybrid System. Sep. Purif. Technol..

[111] Quyet Truong D., Choo Y., Akther N., Roobavannan S., Norouzi A., Gupta V., Blumenstein M., Vinh Nguyen T., Naidu G. (2023). Selective Rubidium Recovery from Seawater with Metal-Organic Framework Incorporated Potassium Cobalt Hexacyanoferrate Nanomaterial. Chem. Eng. J..

[112] Özmal F., Saygılı Canlıdinç R., Erdoğan Y. (2021). Recovery of Rubidium(I) from Boron Industrial Waste with Potassium Cobalt Hexacyanoferrate. Sep. Sci. Technol..

[113] Gao L., Ma G., Zheng Y., Tang Y., Xie G., Yu J., Liu B., Duan J. (2020). Research Trends on Separation and Extraction of Rare Alkali Metal from Salt Lake Brine: Rubidium and Cesium. Solvent Extr. Ion Exch..

[114] Jiang X., Sha Y., Cai R., Shao Z. (2015). The Solid-State Chelation Synthesis of LiNi_1/3_Co_1/3_Mn_1/3_O_2_ as a Cathode Material for Lithium-Ion Batteries. J. Mater. Chem. A Mater. Energy Sustain..

[115] Hillman A.R., Ryder K.S., Ismail H.K., Unal A., Voorhaar A. (2017). Fundamental Aspects of Electrochemically Controlled Wetting of Nanoscale Composite Materials. Faraday Discuss..

[116] Chen W., Xia X.-H. (2007). Highly Stable Nickel Hexacyanoferrate Nanotubes for Electrically Switched Ion Exchange. Adv. Funct. Mater..

[117] Chen W., Tang J., Cheng H.J., Xia X.H. (2009). A Simple Method for Fabrication of Sole Composition Nickel Hexacyanoferrate Modified Electrode and Its Application. Talanta.

[118] Chen S., Dong Y., Wang H., Sun J., Wang J., Zhang S., Dong H. (2022). Highly Efficient and Selective Cesium Recovery from Natural Brine Resources Using Mesoporous Prussian Blue Analogs Synthesized by Ionic Liquid-Assisted Strategy. Resour. Conserv. Recycl..

[119] Sebti E., Besli M.M., Metzger M., Hellstrom S., Schultz-neu M.J., Alvarado J., Christensen J., Doe M., Kuppan S. (2020). Removal of Na^+^ and Ca^2+^ with Prussian Blue Analogue Electrodes for Brackish Water Desalination. Desalination.

[120] Ren Y., Zheng W., Duan X., Goswami N., Liu Y. (2022). Recent Advances in Electrochemical Removal and Recovery of Phosphorus from Water: A Review. Environ. Funct. Mater..

[121] Abdel-Shafy H.I., Morsy R.M.M., Hewehy M.A.I., Razek T.M.A., Hamid M.M.A. (2022). Treatment of Industrial Electroplating Wastewater for Metals Removal via Electrocoagulation 1. Abdel-Shafy, H.I.; Morsy, R.M.M.; Hewehy, M.A.I.; Razek, T.M.A.; Hamid, M.M.A. Treatment of Industrial Electroplating Wastewater for Metals Removal via Electroc. Water Pract. Technol..

[122] Arana Juve J.M., Christensen F.M.S., Wang Y., Wei Z. (2022). Electrodialysis for Metal Removal and Recovery: A Review. Chem. Eng. J..

[123] Qiu Y., Lv Y., Tang C., Liao J., Ruan H., Sotto A., Shen J. (2021). Sustainable Recovery of High-Saline Papermaking Wastewater: Optimized Separation for Salts and Organics via Membrane-Hybrid Process. Desalination.

[124] Garrido B., Cifuentes G., Fredes P., Pino E., Calderón C., Cifuentes-Cabezas M. (2021). Copper Recovery From Ammonia Solutions Through Electro-Electrodialysis (EED). Front. Chem..

[125] Catinean A., Dascalescu L., Lungu M., Dumitran L.M., Samuila A. (2021). Improving the Recovery of Copper from Electric Cable Waste Derived from Automotive Industry by Corona-Electrostatic Separation. Part. Sci. Technol..

[126] Siwal S.S., Kaur H., Deng R., Zhang Q. (2023). A Review on Electrochemical Techniques for Metal Recovery from Waste Resources. Curr. Opin. Green Sustain. Chem..

[127] Madrid F.M.G., Arancibia-Bravo M., Cisterna J., Soliz Á., Salazar-Avalos S., Guevara B., Sepúlveda F., Cáceres L. (2023). Corrosion of Titanium Electrode Used for Solar Saline Electroflotation. Materials.

[128] Madrid F.M.G., Arancibia-Bravo M.P., Sepúlveda F.D., Lucay F.A., Soliz A., Cáceres L. (2023). Ultrafine Kaolinite Removal in Recycled Water from the Overflow of Thickener Using Electroflotation: A Novel Application of Saline Water Splitting in Mineral Processing. Molecules.

